# Autoantigen mRNA‐LNP Vaccination Drives Therapeutic Efficacy in Preclinical Models for Autoimmunity

**DOI:** 10.1002/advs.76382

**Published:** 2026-07-06

**Authors:** Paulien Baeten, Karen Beets, Tessa Schalley, Janne Verreycken, Gayel Duran, Daphne Lintsen, Lisa Schuetz, Xue Zhong, Rinke Nieuwschepen, Melissa Schepers, Brecht Moonen, Jeroen Bogie, Jana Van Broeckhoven, Carlo Heirman, Jurgen Van den Heuvel, Bart Vanderborght, Ismael Varela, Roxanne Nouille, Lotte Jacobs, Jessica Filtjens, Sabah Kasmi, Elise Seynaeve, Veronica Mavrovouna, Jana De Vrieze, Michael A. Brehm, Sandra Maréchal, Sophie Janssens, Florence Lambolez, Niels Hellings, Stefaan De Koker, Bieke Broux

**Affiliations:** ^1^ University MS Center Campus Diepenbeek Diepenbeek Belgium; ^2^ Department of Immunology and Infection Biomedical Research Institute Hasselt University Diepenbeek Belgium; ^3^ etherna immunotherapies NV Niel Belgium; ^4^ Internal Medicine Maastricht University Maastricht Netherlands; ^5^ Department of Neuroscience Biomedical Research Institute Hasselt University Diepenbeek Belgium; ^6^ Department of Psychiatry and Neuropsychology Mental Health and Neuroscience Research Institute Maastricht University Maastricht Netherlands; ^7^ Diabetes Center of Excellence Program in Molecular Medicine University of Massachusetts Chan Medical School Worcester Massachusetts USA; ^8^ VIB‐UGent Center for Inflammation Research Ghent University Zwijnaarde Belgium

**Keywords:** autoimmune diseases, lipid nanoparticles, mRNA therapeutics, tolerizing vaccines

## Abstract

Autoimmune diseases like multiple sclerosis (MS) and type 1 diabetes (T1D) lack therapies that induce durable, antigen‐specific immune tolerance. We investigated whether mRNA lipid nanoparticles (LNPs) encoding disease‐relevant autoantigens could re‐establish immune homeostasis in preclinical models. While mRNA‐LNP microbial vaccines evoke strong effector immune responses, we show that both systemic and intramuscular delivery of MOG_27‐63_ mRNA‐loaded LNPs attenuated disease severity in experimental autoimmune encephalomyelitis (EAE). Antigen‐specific protection was similarly observed in a T1D adoptive transfer model. Therapeutic efficacy achieved using immunostimulatory LNPs challenges the current assumption that tolerogenic mRNA vaccines require immune‐silent LNPs. Furthermore, divergent outcomes between autoantigens and irrelevant antigens suggest that antigen identity determines whether mRNA‐LNPs promote immune tolerance or activation. Mechanistically, optimized LNPs efficiently targeted antigen‐presenting cells (APCs) in the liver and spleen. This promoted a homeostatic APC phenotype and a hyporesponsive CD4^+^ T cell phenotype without inducing regulatory T cells (Tregs). Therefore, autoantigen mRNA was co‐delivered with “immunoregulatory” mRNAs encoding cytokines (IL‐2 mutein) or chemokines (CCL1) known to enhance Treg expansion and recruitment. This co‐delivery further improved clinical outcomes in EAE. Together, these findings demonstrate that systemic and intramuscular treatment with mRNA‐LNPs encoding autoantigens alongside immunoregulatory molecules represents a promising strategy for antigen‐specific immunotherapy in autoimmune diseases.

## Graphical abstract

Systemic and intramuscular delivery of autoantigen mRNA via lipid nanoparticles reprograms antigen‐presenting cells toward a mature, homeostatic state, driving antigen‐specific T cell exhaustion and providing therapeutic efficacy across autoimmune disease models.

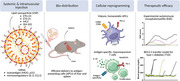

## 1 Introduction

Despite considerable therapeutic advances, chronic autoimmune diseases still pose substantial treatment challenges, and clinical benefit is often coupled to broad immune suppression and associated safety risks. For instance, multiple sclerosis (MS) is characterized by activation of myelin‐reactive T cells and infiltration of immune cells into the central nervous system (CNS), leading to demyelination and, eventually, neurodegeneration. Clinically, MS mostly presents in young adults with relapsing‐remitting episodes of sensory, motor, or visual disturbances. Over time, despite the availability of highly effective treatment options, most patients still transition to a progressive stage marked by accumulating disability. Current therapeutic approaches for MS are based on general modulation of the immune system, and while they effectively reduce relapse rates and MRI lesion activity, they delay disease progression at best and often carry significant adverse effects. Type 1 diabetes (T1D) is a chronic autoimmune disorder characterized by immune‐mediated destruction of the insulin‐producing β cells of the pancreatic islets of Langerhans. Their progressive loss leads to absolute insulin deficiency and chronic hyperglycemia. Without exogenous insulin, patients are at risk of acute metabolic decompensation, including diabetic ketoacidosis, as well as long‐term microvascular complications. Current therapies remain management strategies, such as lifelong exogenous insulin replacement or even transplantation.

Significant efforts are being made to re‐establish antigen‐specific tolerance as a treatment of autoimmune diseases: suppressing pathogenic responses to defined self‐antigens while preserving the capacity to mount protective immunity against foreign antigens. Here, antigen‐specific tolerogenic vaccines offer an attractive approach by aiming to re‐establish immune homeostasis without global immunosuppression. These strategies seek to deliver disease‐relevant autoantigens to antigen‐presenting cells (APCs) in a non‐inflammatory context, promoting T cell anergy, exhaustion, or regulatory T cell (Treg) differentiation. While early clinical efforts using soluble peptide antigens were unsuccessful [1, 2, 3, 4], recent nanoparticle‐based approaches have demonstrated encouraging efficacy in preclinical and early clinical studies (e.g., NCT04602390; NCT04602390; NCT05660109) across multiple autoimmune diseases [5, 6, 7, 8, 9, 10].

mRNA‐based technologies have transformed immunotherapy, as demonstrated by their success in infectious disease vaccines and cancer, where they have been exploited primarily to elicit immunity [11]. In autoimmunity, mRNA‐engineered chimeric antigen receptor (CAR) T cells targeting pathogenic B cells have shown striking efficacy in myasthenia gravis [12], and clinical trials are now underway in rheumatoid arthritis and systemic lupus erythematosus [13]. CAR T cell therapy is also explored in phase I studies for MS (NCT06220201). However, this autologous strategy is constrained by complex manufacturing, costs, and safety concerns (malignancies), limiting its applicability, particularly in early disease. Antigen‐specific tolerogenic vaccines offer an attractive alternative approach. However, a key requirement for this strategy is limiting inflammatory signaling during antigen presentation. Accumulating evidence now indicates that mRNA formulation, nucleoside chemistry, and delivery route can be engineered to support tolerogenic, rather than immunogenic outcomes, in the context of autoimmunity treatment [14]. The versatility of mRNA platforms offers unique advantages for tolerogenic vaccine development, including rapid design, scalable manufacturing, the capability to encode many epitopes, and easy multiplexing of mRNA molecules (e.g., autoantigens and immunomodulatory factors) within lipid nanoparticle (LNP) formulations. However, a central challenge remains: mRNA‐LNPs possess intrinsic adjuvant activity when administered intramuscular (i.m.) [15, 16, 17], raising the question of whether mRNA‐LNP systems can be tuned to induce antigen‐specific tolerance instead of immune activation. In particular, it is unclear how biodistribution to homeostatic APC niches, the maturation state induced in those APCs, and the downstream fate of autoreactive T cells relate to therapeutic efficacy and durability across autoimmune settings.

Here, we demonstrate that mRNA‐LNP formulations previously shown to elicit robust adaptive immune responses against microbial antigens following i.m. vaccination [15, 18, 19] instead promote immune modulation when intravenously (i.v.) or i.m. delivering autoantigens in preclinical models for MS and T1D. Tolerance induction was correlated with a mature, homeostatic APC state, leading to a hyporesponsive T cell phenotype without classical Treg induction. Therefore, we explored co‐encapsulation of mRNA encoding for factors that enhance Treg expansion and function. Indeed, co‐delivery of immunoregulatory mRNA could further delay disease onset or induce disease attenuation. Altogether, these results highlight the substantial potential of LNP‐loaded autoantigen‐specific mRNA, combined with immunoregulatory mRNA co‐payloads, as a promising antigen‐specific therapy for autoimmune diseases, providing a path toward sustained, long‐term tolerance without systemic immunosuppression.

## 2 Results

### 2.1 Presymptomatic and Therapeutic Vaccination With MOG mRNA‐LNPs Alleviates Preclinical Autoimmune Models

To evaluate the potential of mRNA‐LNPs to evoke tolerance against mRNA‐encoded autoantigens, we designed an mRNA encoding MOG_27‐63_, encompassing the immunodominant epitope MOG_35‐55_ of myelin oligodendrocyte protein. We assessed its efficacy in the experimental autoimmune encephalomyelitis (EAE) model, a clinically relevant model of MS. To enhance MHC class II‐mediated antigen presentation to CD4^+^ T cells, the mRNA construct was engineered to include a DC‐Lamp‐derived endosomal targeting domain fused to the MOG peptide (Figure S1), as previously described [20, 21]. Ultrapure, N‐1 methylpseudo‐uridine modified mRNA was used to avoid mRNA‐induced activation of RNA sensors and innate activation. The absence of mRNA‐induced innate activation was demonstrated using the A549‐dual reporter system (Figure S2).

To formulate MOG mRNA in LNPs, we selected S‐Ac7‐Dog (ETG‐23) as the ionizable lipid of choice. LNPs incorporating this lipid have been shown to support robust mRNA expression while eliciting minimal innate immune activation following i.m. [18] and intravitreal administration [22].

**FIGURE 1:**
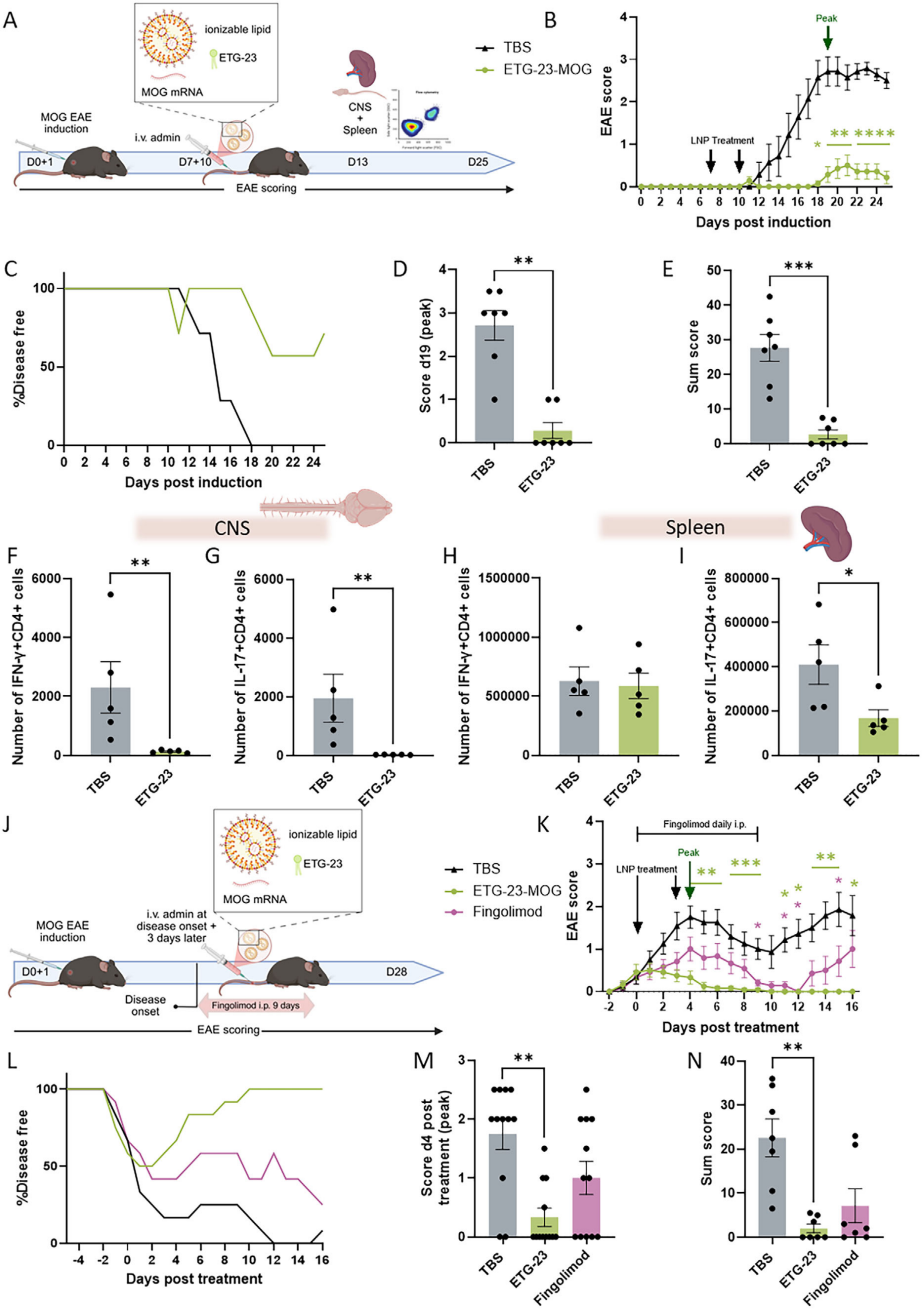
Treatment with ETG‐23‐MOG in EAE prevents disease development and ameliorates disease. MOG_35‐55_ EAE was induced in female C57BL/6 mice. (A–I) At 7 and 10 dpi, mice were i.v. treated with buffer control or ETG‐23 loaded with MOG mRNA (5 µg/dose). (A) Experimental set‐up. (B) Mice were weighed (Figure S3A) and scored daily. Two‐way ANOVA with Tukey's multiple comparison compared to TBS. (C) Incidence of disease‐free mice. (D) Score at peak of disease (19 dpi). (E) Sum of scores. *n* = 7; Mann–Whitney test compared to TBS. (F–I) 13 dpi, immune cells of the CNS (F,G) and spleen (H,I) were isolated and counted. After 4 h of stimulation with PMA, CaI, and Golgiplug, CD4^+^ T cells were analyzed using flow cytometry for IFN‐γ and IL‐17. Gating strategy in Figure S3. *n* = 5; Mann‐Whitney test compared to TBS. (J–N) At disease development, mice were i.v. treated with buffer control or ETG‐23 loaded with MOG mRNA (5 µg/dose). Alternatively, mice were intraperitoneally (i.p.) treated with MS‐approved drug Fingolimod for 9 consecutive days (1 mg/kg). (J) Experimental set‐up. (K) Mice were weighed (Figure S4E) and scored daily. Two‐way ANOVA with Tukey's multiple comparison compared to TBS. (L) Incidence of disease‐free mice. (M) Score at the peak of disease. (N) Sum of scores. *n* = 7‐12; Kruskal‐Wallis test between all groups; ^*^: *p* < 0.05; **: *p* < 0.01; ^***^: *p* < 0.001; ^****^: *p* < 0.0001.

EAE was induced in female mice after subcutaneous injection with MOG_35‐55_ emulsified in complete Freund's adjuvant containing *Mycobacterium tuberculosis*, and intraperitoneal (i.p.) injection of pertussis toxin (PTX) immediately after immunization and on day 1. This induces a CD4^+^ T cell‐mediated autoimmune response against myelin, resulting in demyelination and neurological impairment. Neurological deficits were evaluated daily using a standard five‐point scale (0: no symptoms; 1: limp tail; 2: weakness of hind legs; 3: complete paralysis of hind legs; 4: complete hind and partial front leg paralysis; 5: death). Mice were i.v. treated 7 and 10 days after MOG_35‐55_ EAE induction, before onset of clinical symptoms (Figure 1A–I). Mice treated with TBS developed a typical disease course, while mice treated with ETG‐23 loaded with MOG mRNA (ETG‐23‐MOG) showed a significant delay in disease onset and severity (Figure 1B,C). Both the clinical score at the peak of disease and the sum of scores were significantly reduced after treatment with ETG‐23‐MOG compared to TBS (Figure 1D,E). Three days after the last treatment, immune cells derived from the spleen and CNS were immunophenotyped using flow cytometry. Overall, these data showed an almost complete abolishment of CNS lymphocyte infiltration in ETG‐23‐MOG‐treated mice compared to TBS‐treated mice (Figure S3B–D). Disease‐associated IFN‐γ‐ and IL‐17‐secreting CD4^+^ T cells were virtually absent in the CNS of ETG‐23‐MOG‐treated mice, while only IL‐17‐secreting CD4^+^ T cells were reduced in the spleen, compared to control mice (Figure 1F–I). No induction of CD25^+^ CD4^+^ Tregs was observed in the CNS or spleen in response to mRNA‐LNP treatment (Figure S3B–D).

Next, we tested i.v. mRNA‐LNP administration in an early therapeutic set‐up of EAE, which is more relevant to clinical MS treatment schemes. Herein, treatment was started once mice developed disease, and compared to a standard‐of‐care treatment, fingolimod (daily i.p. treatment for 9 days; Figure 1J–N). Mice treated with ETG‐23‐MOG showed an immediate suppression of disease development compared to TBS, and mice remained disease‐free until the end of the experiment (Figure 1J–N). Indeed, both disease severity at the peak (Figure 1M) and the sum of scores (Figure 1N) were significantly decreased using ETG‐23‐MOG treatment compared to TBS. To verify the antigen‐specificity of the therapeutic effect, an irrelevant mRNA (i.e., firefly luciferase; Fluc) was included as well. ETG‐23‐Fluc did not impact disease severity (Figure S4A‐E). Fingolimod temporarily reduced symptoms, however, not as fast or effective as ETG‐23‐MOG (Figure 1K). Even more, after treatment cessation, fingolimod‐treated mice quickly relapsed (Figure 1K). Taken together, these data indicate that, in this preclinical model and treatment regimen, ETG‐23‐MOG demonstrated greater efficacy than fingolimod, in an antigen‐specific manner.

**FIGURE 2:**
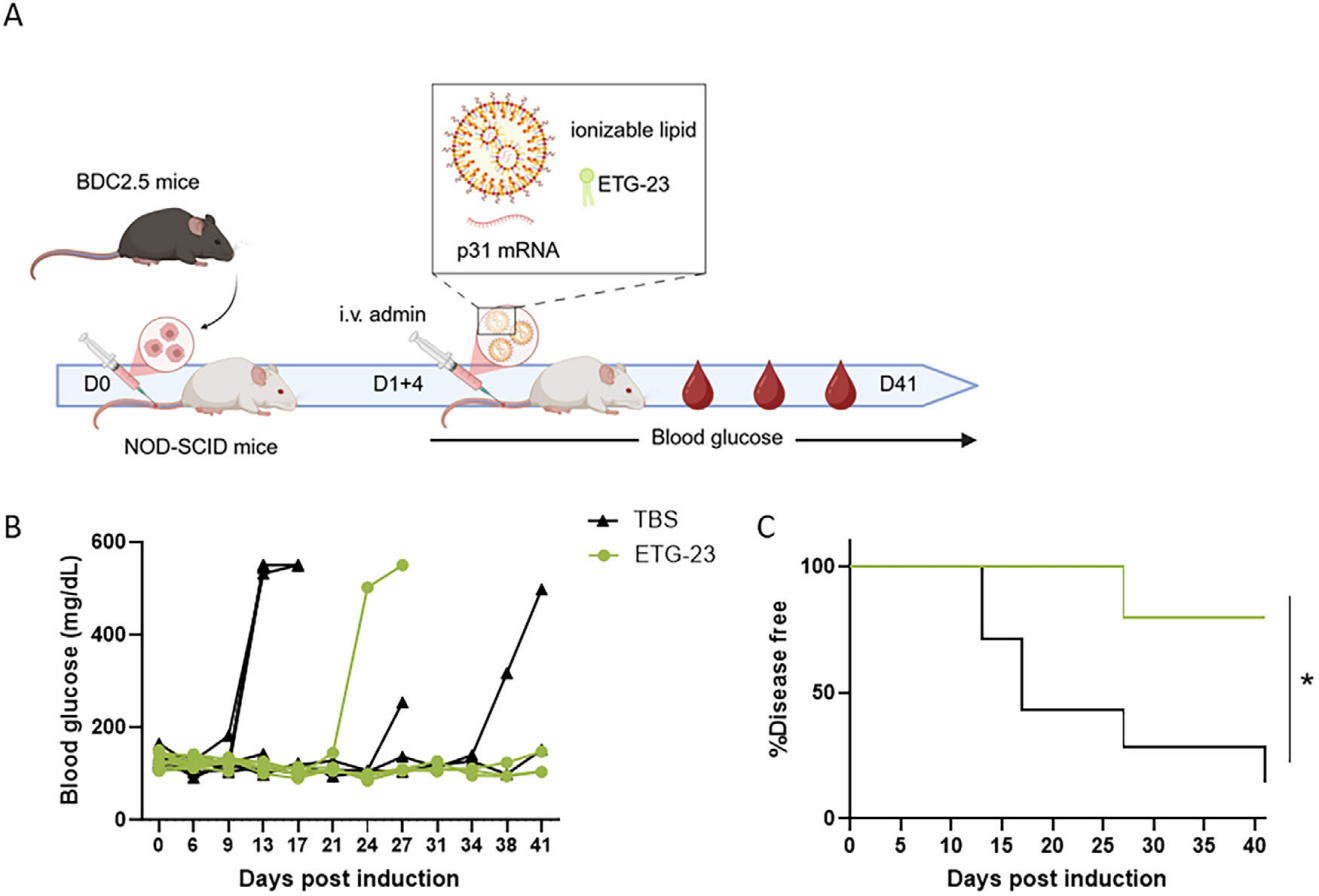
Treatment with ETG‐23‐p31 prevents the development of T1D. NOD‐SCID mice received CD4^+^ T cells from BDC2.5 transgenic mice to induce diabetes. 1 and 4 days later, recipient mice received i.v. treatment with ETG‐23‐p31 (5 µg/dose) or buffer control. (A) Experimental set‐up. (B) Blood glucose levels were measured twice a week, and mice were weighed regularly (Figure S5A). (C) Incidence of disease‐free mice. *n* = 5–10. A Logrank (Mantel‐Cox) test was performed to compare the groups and resulted in *p* = 0.03.

To understand whether this approach would also be effective in another preclinical autoimmune model, we investigated the therapeutic potential of i.v. mRNA‐LNP administration in the context of T1D. Here, NOD‐SCID mice received activated, antigen‐specific CD4^+^ T cells from BDC2.5 transgenic mice, which express a pancreatic autoantigen (p31)‐specific T cell receptor [23], to induce T1D. Following transfer, these antigen‐specific CD4^+^ T cells mediate an autoimmune response against pancreatic β cells, resulting in progressive loss of insulin‐producing β cells and thus loss of glycemic control. One and four days later, recipient mice received i.v. treatment with ETG‐23‐p31 or TBS (Figure 2A–C). Then, blood glucose levels were measured twice a week to evaluate glucose tolerance. Almost all TBS‐treated mice quickly demonstrated increased glucose levels. By day 30, 75% of the mice developed disease (Figure 2B,C and Figure S5A). In contrast, all but one ETG‐23‐p31‐treated mice showed preserved glucose tolerance up to 41 days after disease induction (Figure 2B,C). In addition, histological examination revealed that ETG‐23‐p31 treatment preserved pancreatic β cells, as evidenced by robust insulin staining and intact islet morphology, whereas the TBS‐treated group exhibited marked islet infiltration and reduced insulin presence (Figure S5B–D). Insulitis was quantified in H&E‐stained pancreatic sections using a 0–4 scoring system (Figure S5C,D). Most islets in TBS displayed severe insulitis (scores 3–4), whereas islets in ETG‐23‐p31 predominantly showed little to no infiltration (scores 0–1), indicating reduced autoimmune‐mediated islet damage after mRNA‐LNP treatment. Furthermore, no impact on hyperglycemia was observed after treatment with ETG‐23‐Fluc, demonstrating that therapeutic efficacy is not due to the LNP components, but rather the autoantigen mRNA (Figure S5E). In conclusion, antigen‐specific mRNA‐LNP treatment almost completely prevents the development of hyperglycemia, showing its therapeutic potential across different autoimmune pathologies.

### 2.2 The Immune‐Stimulatory Lipid ETS‐21 Exhibits Therapeutic Activity in Established EAE After Systemic and Intramuscular Delivery

The ionizable lipid is a critical component of LNP formulations, as its chemical properties strongly influence mRNA expression, biodistribution, and the magnitude of innate immune activation [14]. To determine whether a low inflammatory profile of ionizable lipids [18] is essential for the therapeutic efficacy of MOG mRNA‐LNP immunization in EAE, we evaluated two alternative ionizable lipids known to elicit stronger innate immune stimulation. MC3 [24] is a structurally distinct ionizable lipid used in the clinically approved formulation Onpattro that has been shown to induce higher levels of inflammatory cytokines and chemokines (IL‐6, CCL2, and CXCL1) after i.m. injection [18, 25]. ETS‐21 (Ac6e‐ETH‐DHDA) is an ionizable lipid structurally related to ETG‐23 that promotes markedly enhanced innate and adaptive immune responses compared with ETG‐23 upon i.m. vaccination [15, 18]. Given the strong immune‐stimulatory properties of ETS‐21 in an i.m. vaccination context [15], we hypothesized that this lipid might be less effective or even detrimental in a tolerizing vaccine context.

**FIGURE 3:**
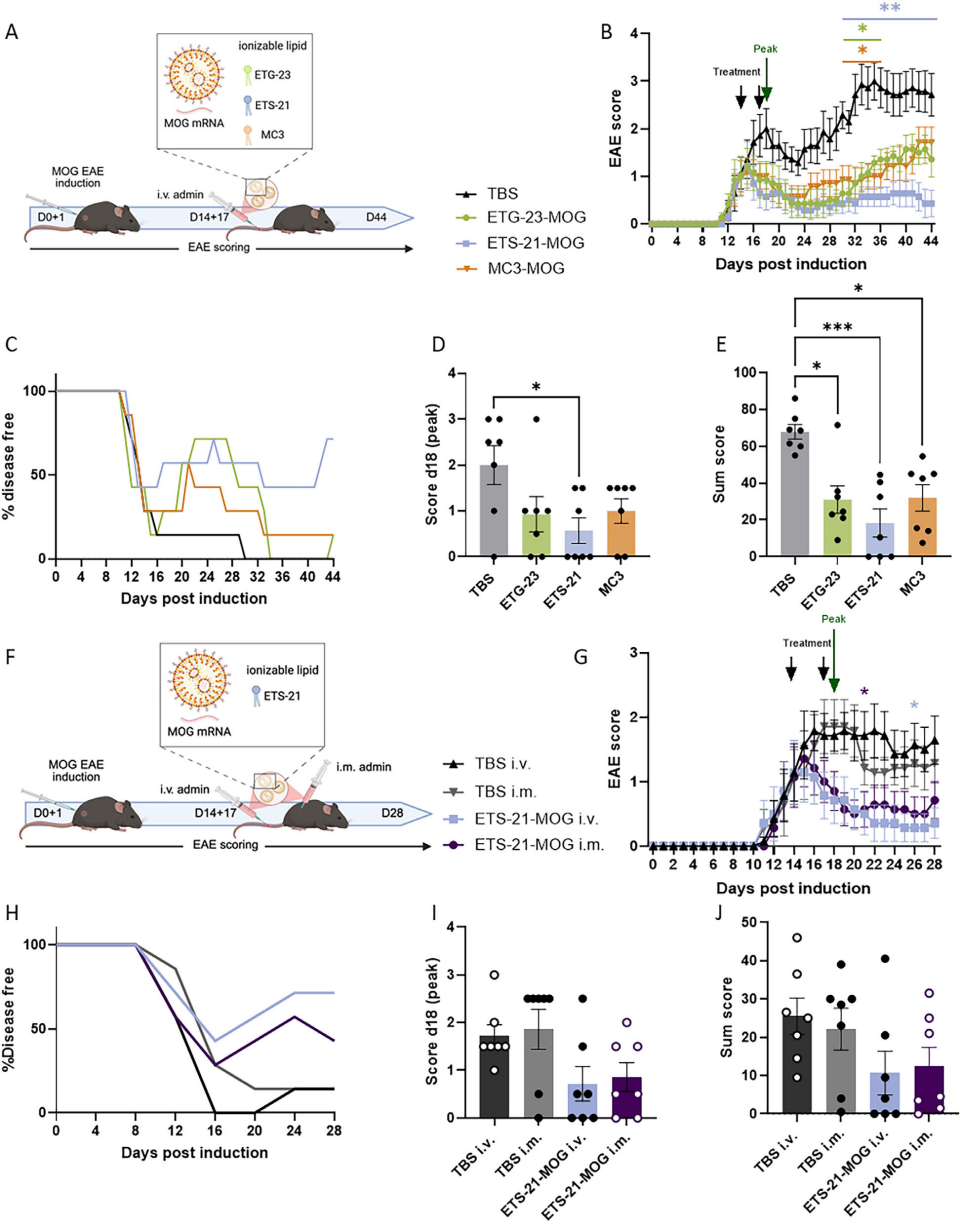
Immune‐stimulatory lipid ETS‐21 exhibits therapeutic activity in established EAE after systemic and i.m. delivery. (A‐E) MOG_35‐55_ EAE was induced in female C57BL/6 mice. On days 14 and 17 after disease induction, mice were i.v. treated with buffer control or different LNPs loaded with MOG mRNA (5 µg/dose). (A) Experimental set‐up. (B) Mice were weighed (Figure S4F) and scored daily. Two‐way ANOVA with Tukey's multiple comparison compared to TBS. (C) Incidence of disease‐free mice. (D) Score at peak of disease (18 dpi). (E) Sum of scores. *n* = 7; (F–J) MOG_35‐55_ EAE was induced in female C57BL/6 mice. On days 14 and 17 after disease induction, mice were i.v. or i.m. treated with buffer control or ETS‐21 loaded with MOG mRNA (5 µg/dose). (F) Experimental set‐up. (G) Mice were weighed (Figure S4G) and scored daily. Two‐way ANOVA with Tukey's multiple comparison compared to TBS. (H) Incidence of disease‐free mice. (I) Score at peak of disease (18 dpi). (J) Sum of scores. *n* = 6‐7; Kruskal‐Wallis test compared to TBS; ^*^: *p* < 0.05; ^**^: *p* < 0.01; ^***^: *p* < 0.001.

EAE mice were i.v. treated with these three different LNPs loaded with MOG mRNA at 14 and 17 days post‐induction (Figure 3A), of which tolerability was assessed, and no toxicity was found (Figure S6). These timepoints were chosen to more closely reflect the clinical presentation of MS patients, who usually only seek medical attention when the disease has fully manifested. All i.v. administered mRNA‐LNPs significantly reduced disease severity (Figure 3B–E). Surprisingly, treatment with MOG mRNA formulated in ETS‐21, specifically optimized to evoke maximal effector T and B cell responses upon i.m. administration [15, 18], showed superior efficacy to ETG‐23‐MOG or MC3‐MOG, characterized by a prolonged disease suppression (Figure 3B–E). Even more, half of the ETS‐21‐MOG‐treated mice remained disease‐free until the end of the experiment (Figure 3C–E), which was in contrast to ETG‐23‐MOG or MC3‐MOG, where only a few mice remained disease‐free. Compared to an earlier therapeutic setting, this delayed treatment with ETG‐23 was found to be less effective. Together, these results showed that ETS‐21‐MOG treatment attenuated ongoing EAE more efficiently than ETG‐23‐MOG or the clinically approved LNP, MC3‐MOG, as demonstrated by the prolonged therapeutic effect. These data challenge the current dogma that LNPs need to be specifically designed for tolerogenicity, but rather can be repurposed from their use in vaccination against infectious diseases.

To assess whether the route of administration influences the tolerogenic effect of antigen‐loaded LNPs, ETS‐21 and the clinically validated SM‐102 formulation, best known for Moderna's COVID‐19 vaccine [19], were administered either i.v. or i.m. in EAE mice on days 14 and 17 post‐induction (Figure 3F). Both i.v. and i.m. administration of ETS‐21‐MOG reduced disease severity compared with TBS‐treated controls (Figure 3G–J). Despite the well‐established immunostimulatory properties of SM‐102 mRNA‐LNPs in infectious disease vaccines, SM‐102‐MOG induced comparable disease amelioration when administered via either route (Figure S4H). These findings indicate that therapeutic efficacy, even of immunogenic LNPs, is independent of the administration route and instead is suggested to be driven by the nature of the delivered antigen cargo. Of importance, these observations show that the same LNP formulations used in vaccination settings can also promote antigen‐specific tolerance when used to deliver autoantigens in autoimmune disease contexts.

### 2.3 LNP Administration Generates Mature, Homeostatic APCs

To elucidate the mechanisms underlying mRNA‐LNP vaccination and to understand why certain LNP formulations are superior at inducing therapeutic efficacy, we analyzed the biodistribution, cellular tropism, and APC phenotype following i.v. administration. To assess mRNA biodistribution, LNPs encapsulating Fluc mRNA were administered i.v. and analyzed by whole‐body in vivo imaging system (IVIS). All three mRNA‐LNP formulations (ETG‐23, ETS‐21, and MC3) mediated robust luciferase expression (Figure S7A). Ex vivo imaging revealed predominant expression in the liver and spleen, with ETS‐21 inducing markedly higher signal intensity than ETG‐23 or MC3 (Figure S7A–C).

**FIGURE 4:**
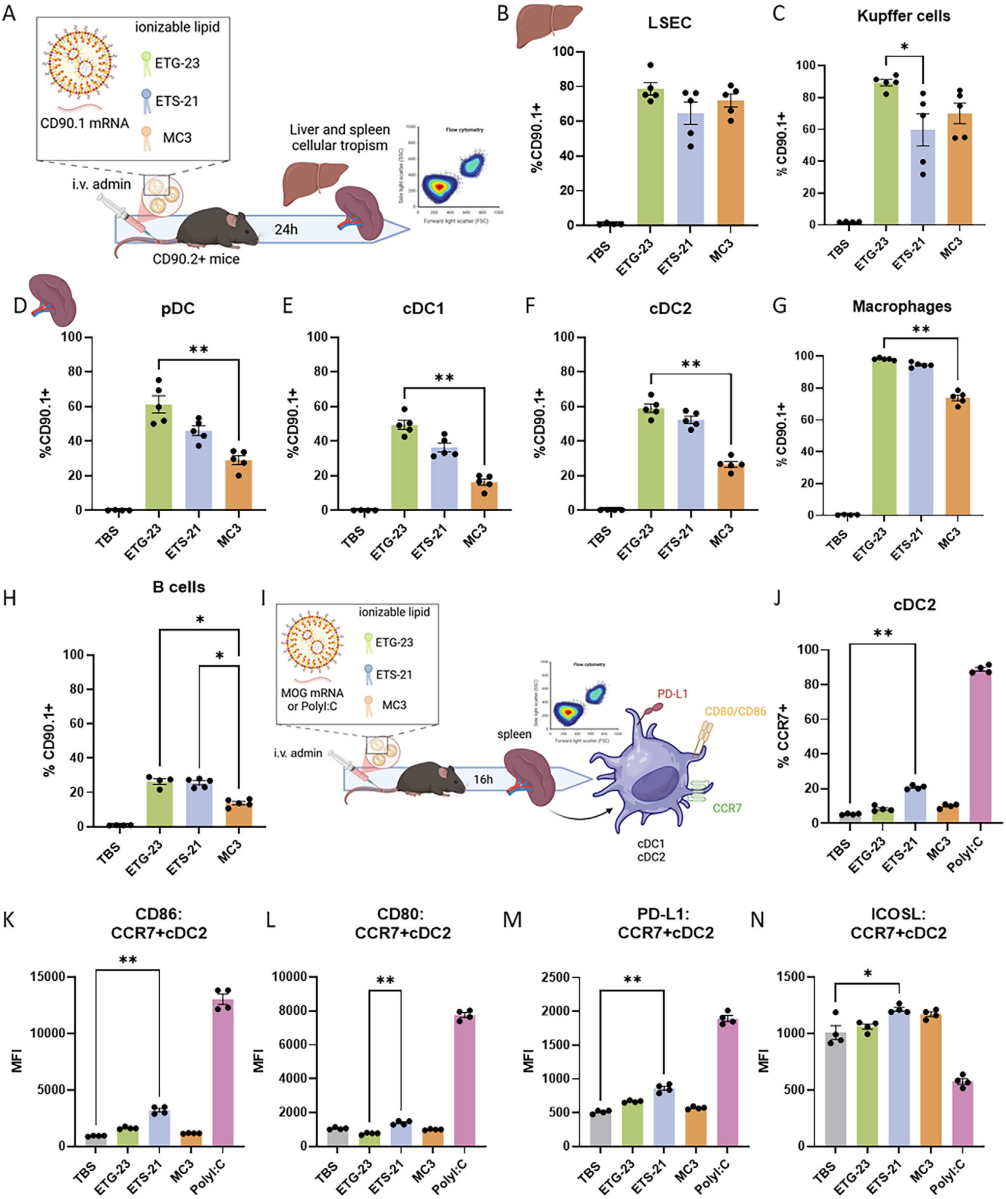
mRNA‐LNPs target antigen‐presenting cells of liver and spleen and induce a mature, homeostatic phenotype. (A–G) CD90.2 C57BL/6 mice were i.v. injected with different LNPs loaded with CD90.1 mRNA (20 µg/dose). After 24 h, cells were isolated from the liver (B,C) and spleen (D–H) and investigated using flow cytometry. Gating strategy in Figure S8. *n* = 3‐5, Kruskal–Wallis test compared within LNP groups. (I–N) Mice were i.v. injected with different LNPs loaded with MOG mRNA or PolyI:C in ETG‐23 (5 µg/dose). After 16 h, co‐stimulatory markers CD86 and CD80, immunogenic marker PD‐L1, and homeostatic molecule ICOSL in CCR7^+^ cDC were investigated in the spleen using flow cytometry. Gating strategy in Figure S13. Kruskal–Wallis test compared within groups excluding PolyI:C. *n* = 4; ^*^: *p* < 0.05; ^**^: *p* < 0.01; ^***^: *p* < 0.001; LSEC: liver sinusoidal endothelial cells; pDC: plasmacytoid dendritic cells; cDC: conventional dendritic cells: PolyI:C: polyinosinic‐polycytidylic acid.

To quantify mRNA delivery at the cellular level, we i.v. administered LNPs encoding CD90.1 mRNA into CD90.2^+^ C57BL/6 mice, enabling sensitive detection of CD90.1 expressing cells using isoform‐specific antibodies (Figure 4A–H). Gating strategies and representative flow cytometry plots are shown in Figure S8. Across all formulations, LNP‐mediated mRNA delivery was efficient in APC populations. In the liver, 40%–80% of liver sinusoidal endothelial cells (LSECs) and Kupffer cells expressed the encoded payload (Figure 4B,C). Moreover, neutrophils, macrophages, monocytes, and B cells were also efficiently transfected (Figure S9A). In the spleen, substantial fractions of dendritic cells (DCs), including cDC1, cDC2, and pDC subsets, as well as macrophages and to a lesser extent B cells, were transfected (Figure 4D–H). While overall transfection patterns were comparable among LNPs, ETG‐23 and ETS‐21 tended to transfect a higher proportion of splenic DCs than MC3 (Figure 4D–F). DCs play a central role in orchestrating immunity and tolerance. During infection, engagement of pattern recognition receptors induces DC maturation characterized by upregulation of MHC class II, co‐stimulatory molecules, and CCR7, alongside robust inflammatory cytokine production, driving effector T cell differentiation. In contrast, DCs can also undergo homeostatic maturation in the absence of microbial cues [26]. This process involves moderate upregulation of co‐stimulatory molecules and minimal inflammatory cytokine secretion [26]. These DCs are thought to promote peripheral tolerance through T cell anergy, exhaustion, deletion, or regulatory differentiation [27]. Given this critical role of DCs, we next assessed the impact of distinct MOG mRNA‐LNP treatments on the maturation status of splenic DCs (Figure 4I). As a benchmark for immunogenic DC activation, we delivered the TLR3 agonist polyI:C using ETG‐23. Compared to TBS, ETS‐21 significantly increased the frequency of CCR7^+^ cDC1 and cDC2 subsets (Figure 4J and Figure S9B). Within the mature CCR7^+^ DC population, we examined expression of co‐stimulatory molecules CD80 and CD86, as well as the immunogenic molecule PD‐L1 [28] and homeostatic marker ICOSL. Whereas ETG‐23 and MC3 induced minimal changes within mature DC, ETS‐21 significantly upregulated CD80 and CD86 compared to TBS; albeit to a substantially lower extent than polyI:C (Figure 4K,L and Figure S9B). Even more, expression of the immunogenic marker PD‐L1 and homeostatic marker ICOSL was significantly induced by ETS‐21 compared to TBS (Figure 4M,N and Figure S9B). Similarly, in the APC cells of the liver, CD80 and PD‐L1 expression by Kupffer cells was elevated after mRNA‐LNP treatment (Figure S9C).

To assess whether ETS‐21 administration induced an acute systemic immune response, an earlier time point was included (Figure S10). In contrast to 16 hpi, ETS‐21 did not increase the frequency of CCR7^+^ cDC subsets 2 h after administration (Figure S10A). Furthermore, only very modest changes in the expression of CD86, CD80, or PD‐L1 were observed in CCR7^+^ cDC subsets 2 hpi, similar to 16 hpi (Figure S10B,C). Consistent with these findings, serum cytokine and chemokine levels remained largely unchanged 5 hpi (Figure S6A). Together, these data indicate that ETS‐21 administration does not elicit a pronounced acute systemic inflammatory response following i.v. injections.

Additionally, B cells in the spleen were also investigated following ETS‐21 administration, as they are another potential APC in this context. At 2 hpi, no differences could be detected between TBS and ETS‐21 (Figure S10D). In contrast, at 16 hpi, ETS‐21 significantly increased the expression of co‐stimulatory molecule CD86, as well as immunogenic marker PD‐L1 and homeostatic marker ICOSL. Importantly, this induction of CD86 and PD‐L1 remained moderate compared to polyI:C (Figure S10D). These findings indicate that ETS‐21‐treated B cells maintain a predominantly homeostatic phenotype, with only limited acquisition of markers typically associated with inflammatory antigen presentation.

Collectively, these results demonstrate that LNPs, even when they are designed to be immunostimulatory, promote the expansion of a DC population exhibiting a phenotype characteristic of homeostatic DC maturation when they are loaded with autoantigen mRNA. This observation is consistent with recent studies showing that cholesterol‐rich LNPs can induce homeostatic DC maturation through an LXR‐dependent mechanism [26]. Notably, the LNP that produced the highest upregulation of CD80, CD86, and PD‐L1 also achieved the greatest therapeutic efficacy, suggesting that a limited upregulation of these immunogenic molecules is actually beneficial for tolerance induction in the context of autoimmunity.

### 2.4 Systemic mRNA‐LNP Vaccination Drives Autoantigen‐Specific T Cells Toward an Exhausted and Anti‐Inflammatory Phenotype

**FIGURE 5:**
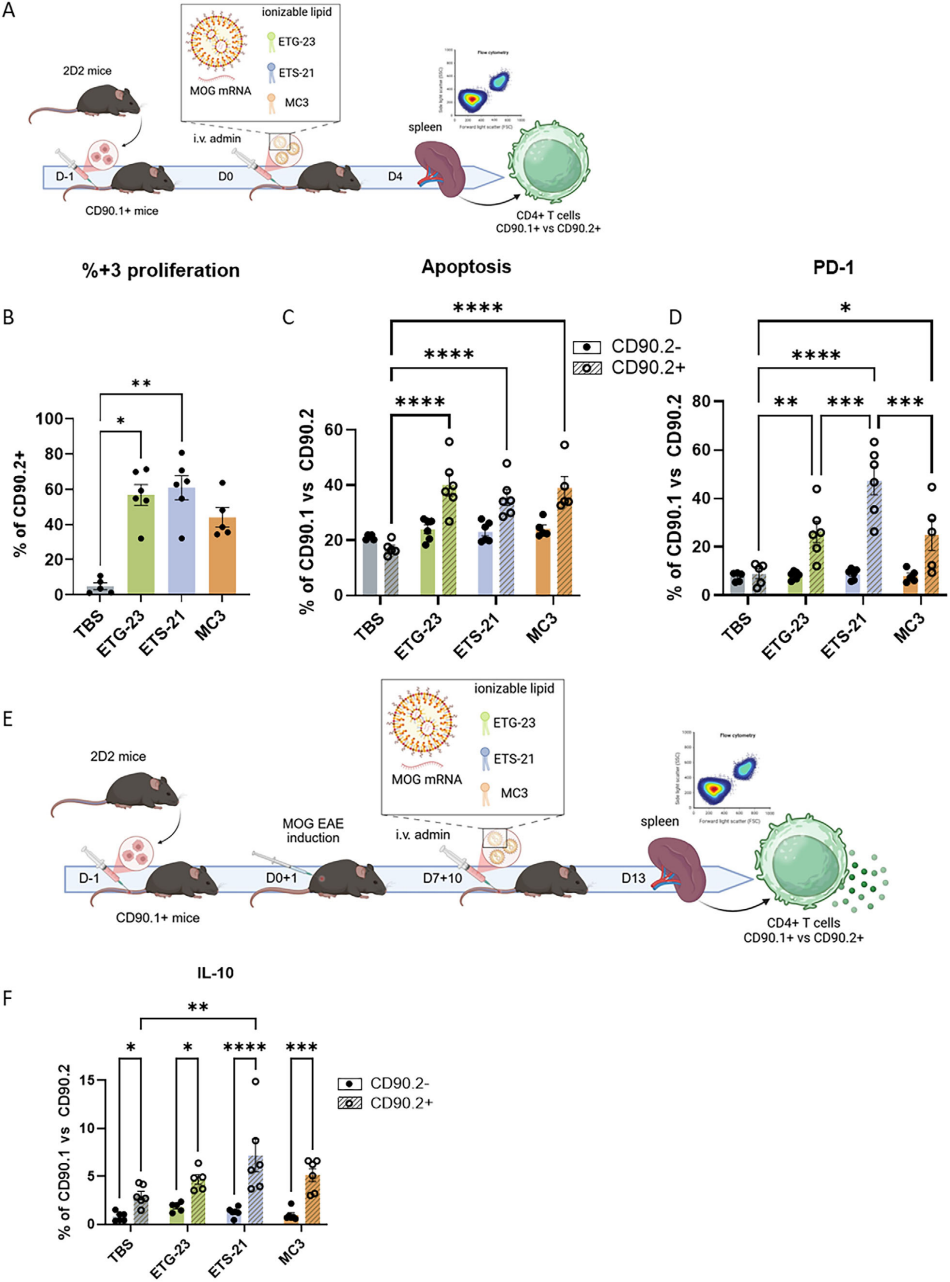
Induction of exhaustion and anti‐inflammatory markers in antigen‐specific CD4^+^ T cells by MOG mRNA‐LNPs. (A‐D) CD4^+^ T cells were isolated from 2D2 transgenic mice (CD90.2^+^ background), labeled with CellTrace Violet and i.v. injected in recipient mice (CD90.2^−^ background). The next day, mice were i.v. injected with buffer control or with different LNPs loaded with MOG mRNA (5 µg/dose). After 4 days, splenocytes and CD4^+^ T cells were isolated and studied using flow cytometry. Gating strategy in Figure S14. (A) Experimental set‐up. (B) Analysis of CD90.2^+^ donor cell proliferation in response to treatment. Proliferation of more than three divisions were evaluated. *n* = 5‐6. Kruskal‐Wallis test within groups. (C‐D) Analysis of %apoptotic^+^ cells stained with ApoTracker (C) or %PD‐1^+^ cells (D) of both CD90.2 positive and negative cells. *n* = 5‐6. Two‐way ANOVA test with Tukey's multiple comparison, only showing differences between LNPs. (E,F) CD4^+^ T cells were isolated from 2D2 transgenic mice (CD90.2^+^ background), and i.v. injected in recipient mice (CD90.2^−^ background). The next day, MOG_35‐55_ EAE was induced in recipient mice. On days 7 and 10, mice were i.v. injected with buffer control or different LNPs loaded with MOG mRNA (5 µg/dose). After 3 days, splenocytes were isolated and studied using flow cytometry after 4 h stimulation with PMA, CaI, and GolgiPlug. Gating strategy in Figure S14. (E) Experimental set‐up. (F) Analysis of anti‐inflammatory marker IL‐10 within both CD90.2‐positive and negative cells. *n* = 5‐6; Two‐way ANOVA test with Tukey's multiple comparison comparing all conditions; ^*^: *p* < 0.05; ^**^: *p* < 0.01; ^***^: *p* < 0.001; ^****^: *p* < 0.0001.

Next, we investigated whether mRNA expression by APCs resulted in functional antigen presentation and antigen‐specific T cell activation. To enable robust tracking of antigen‐specific responses, CD4^+^ T cells from 2D2 transgenic mice, which express a MOG_35‐55_‐specific T cell receptor in a CD90.2 background, were used [29]. These CD4^+^ T cells were labeled with CellTrace Violet and i.v. injected into CD90.2^−^ wild‐type recipient mice. This model allows easy and durable identification and discrimination of transferred MOG‐specific (CD90.2^+^) T cells from polyclonal (CD90.2^−^) T cells. After 24 h, recipient mice received LNPs loaded with MOG mRNA via i.v. administration. Four days later, proliferation of antigen‐specific splenic CD90.2^+^ T cells was determined using flow cytometry (Figure 5A–D). ETG‐23‐MOG and ETS‐21‐MOG induced strong 2D2 T cell proliferation compared to TBS, demonstrating superior functional antigen presentation by APCs compared to MC3‐MOG (Figure 5B). Additionally, compared to the recipient's endogenous CD90.2^−^ T cells, all MOG‐specific CD90.2^+^ T cells showed increased levels of apoptosis (ETG‐23‐MOG: *p* < 0.0001; ETS‐21‐MOG: *p* < 0.001; MC3‐MOG: *p* < 0.001) and exhaustion (PD‐1; ETG‐23‐MOG: *p* < 0.001; ETS‐21‐MOG: *p* < 0.0001; MC3‐MOG: *p* < 0.01) after mRNA‐LNP treatment (Figure 5C,D). All three mRNA‐LNPs induced enhanced levels of apoptosis and PD‐1 of CD90.2^+^ T cells compared to TBS (Figure 5C,D). Moreover, ETS‐21‐MOG triggered significantly higher levels of PD‐1 compared to other LNPs (Figure 5D). Together, these findings suggest that i.v. administered mRNA‐LNPs successfully induce antigen‐specific T cell proliferation, followed by the induction of both apoptosis and exhaustion. A similar induction of anergy could also be observed in the preclinical model for T1D. Here, immune cells from the blood, spleen, and pancreas were analyzed using flow cytometry (Figure S11A). At 8 dpi, a clear CD4^+^ T cell expansion with ETG‐23‐p31 could be observed in all organs compared to TBS (Figure S11A). At 30 dpi, these CD4^+^ T cells were more anergic as presented by an increased expression of PD‐1 after ETG‐23‐p31 treatment (Figure S11A).

To obtain more detailed information about the induced CD4^+^ T cell phenotype during neuroinflammation, we performed a similar experiment in the context of active immunization (Figure 5E,F and Figure S11B,C). Hereto, CD4^+^ T cells from 2D2 transgenic mice were i.v. injected into CD90.2^−^‐recipient mice. After 24 h, recipient mice received active MOG_35‐55_ EAE induction. After 7 and 10 days, recipient mice were i.v. injected with TBS or different LNPs loaded with MOG mRNA. Three days after the last treatment, the phenotype of splenic CD90.2^+^ T cells was determined using flow cytometry (Figure 5F and Figure S11B,C). Strikingly, independent of treatment regimen, antigen‐specific donor T cells acquired an exhausted phenotype compared to the recipient's endogenous CD90.2^−^ T cells regarding increased expression of exhaustion markers PD‐1, GITR, and CD73 (Figure S11B). In a similar way, expression of anti‐inflammatory markers IL‐10 and FOXP3 was increased in CD90.2^+^ T cells compared to CD90.2^−^ T cells (Figure 5F and Figure S11C). This suggests that EAE induction by itself has a substantial impact on antigen‐specific T cells, which is not further impacted by therapeutic vaccination with MOG mRNA‐LNPs. Nonetheless, we did find elevated levels of IL‐10 in antigen‐specific CD90.2^+^ T cells in ETS‐21‐MOG‐treated mice compared to TBS (Figure 5F).

Altogether, these data indicate that i.v. administered LNPs carrying autoantigen mRNA resulted in antigen‐specific T cell proliferation, apoptosis induction, and an exhausted, anti‐inflammatory T cell phenotype. As ETS‐21 was found to significantly induce anti‐inflammatory T cells, combined with its superior therapeutic effect, ETS‐21 was therefore prioritized in subsequent experiments.

### 2.5 Co‐Delivery of Autoantigen and Immunoregulatory mRNA Enhances EAE Disease Attenuation

To assess whether long‐term tolerance could be induced by systemic vaccination with MOG mRNA, we designed an EAE experiment with prophylactic mRNA‐LNP treatment. Long‐term tolerance requires the induction of antigen‐specific Tregs, however, this could not be observed in our previous studies (Figure S3). To boost Treg expansion, we decided to co‐deliver MOG mRNA with mRNAs encoding for cytokines or chemokines known to promote Treg proliferation and/or recruitment (“immunoregulatory” mRNAs). Mice were i.v. injected with MOG mRNA combined with the immunoregulatory mRNA (1:1), an irrelevant mRNA (eGFP) or TBS at 10 and 7 days before active MOG_35‐55_ EAE induction. We explored two different mRNAs: IL‐2 and CCL1. IL‐2 is a crucial cytokine that promotes both effector T cells (driving immune responses) and Treg (suppressing autoimmunity) proliferation, but Tregs are far more sensitive to low doses due to their high‐affinity IL‐2 receptor. To further increase specificity for Tregs, we used a mutated IL‐2 sequence (IL‐2 mutein) with reduced affinity for the IL‐2Rβγ receptor expressed on effector T cells [30, 31]. In EAE, de Picciotto et al. [30] showed that LNP delivery of human IL‐2 mutein ameliorates disease via Tregs. Similarly, Khoryati et al. [31] demonstrated attenuation of disease in a T1D model using mouse IL‐2 mutein. In addition to IL‐2 mutein, we also explored CCL1 mRNA. Tregs express chemokine receptor CCR8, and Barsheshet et al. [32] demonstrated that murine CCL1 triggered the differentiation and expansion of CCR8^+^ Tregs, thereby reducing disease severity in the EAE model.

**FIGURE 6:**
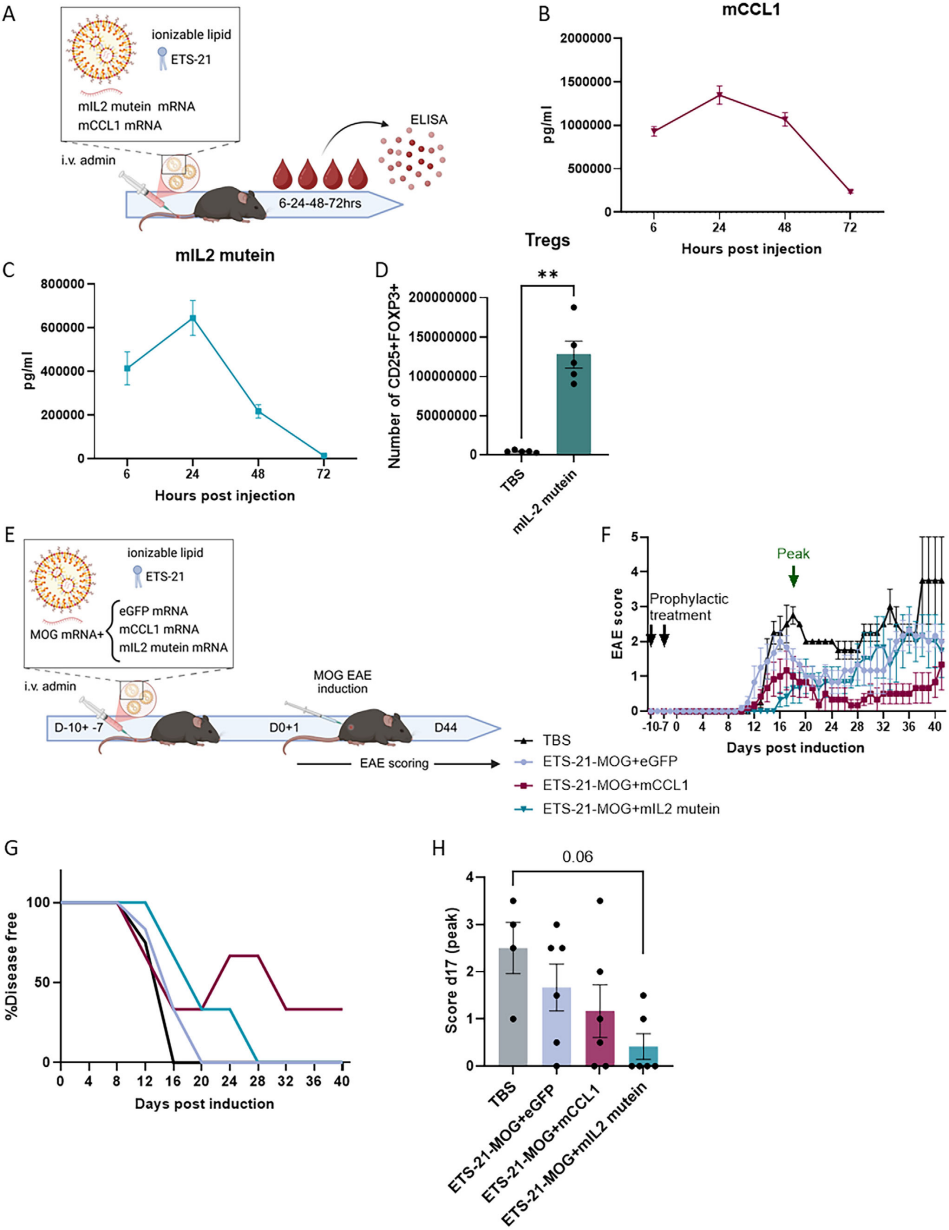
Prophylactic treatment of MOG mRNA combined with robust immunoregulatory mRNA can further delay disease development and ameliorate disease severity. (A–C) Mice were i.v. injected with ETS‐21 loaded with IL‐2 mutein or CCL1 mRNA (5 µg/dose). Serum was collected after 6, 24, 48, or 72 h post‐injection and protein concentrations of IL‐2 mutein or CCL1 were evaluated using ELISA. *n* = 5. (D) FOXP3 reporter mice were i.v. injected with ETS‐21 loaded with IL‐2 mutein mRNA (5 µg/dose) or buffer controls. Spleen was isolated, and immune cells from the spleen were isolated, and Treg numbers were studied using flow cytometry. Gating strategy in Figure S12A. Kruskal‐Wallis test; *n* = 4‐5. (E–G) Female C57BL/6 mice were i.v. injected with ETS‐21 loaded with MOG mRNA with or without immunoregulatory mRNA (5 µg/dose) or buffer control at 10 and 7 days before active MOG_35‐55_ EAE induction. (E) Experimental set‐up. (F) Mice were weighed (Figure S12D) and scored daily. Two‐way ANOVA with Tukey's multiple comparison compared between shown groups. (G) Incidence of disease‐free mice. Two‐way ANOVA with Tukey's multiple comparison compared between shown groups. Kruskal–Wallis test within shown groups; *n* = 3‐6; Data from this Figure and Figure S12 are from the same experiment. Kruskal–Wallis test within the shown groups. ^*^: *p* < 0.05.

First, a robust presence of CCL1 and IL‐2 mutein in the serum of mice after one systemic injection of mRNA‐LNPs was detected (Figure 6A–C). In addition, we demonstrated that in FOXP3 reporter mice, the number of CD25^+^FOXP3^+^ Tregs, but not CD25^−^FOXP3^−^ effector T cells, was significantly increased in the spleen after i.v. delivery of IL‐2 mutein (Figure 6D and Figure S12B). After prophylactic treatment of EAE mice (Figure 6E–H), ETS‐21 loaded with MOG mRNA without immunoregulatory mRNA could only moderately attenuate the disease course in comparison to TBS (Figure 6F–H). Due to the lack of Treg induction in previous experiments, this observation was not unexpected. In contrast, the combination of MOG mRNA with IL‐2 mutein mRNA delayed disease onset and decreased disease severity at the beginning of the disease (Figure 6F–H). However, as the disease progressed, all mice developed a disease similar to MOG mRNA (Figure 6F–H). Importantly, the combination of MOG mRNA with CCL1 mRNA attenuated the disease score mildly at the beginning of the disease (Figure 6F–H). However, clinical symptoms were suppressed as the disease progressed, and several mice remained disease‐free (Figure 6F–H).

To understand whether this effect was mediated through the induction of Tregs, we immunophenotyped the spleen at the peak of disease. Both the combination of autoantigen with IL‐2 mutein or CCL1 mRNA yielded a minor induction of CD25^+^ Tregs compared to MOG mRNA alone (Figure S12C). Of note, ETS‐21 loaded with either IL‐2 mutein or mCCL1 mRNA without autoantigen mRNA exacerbated the peak of disease or triggered the premature onset of disease, respectively, compared to MOG mRNA and TBS (Figure S12E,F). Together, these data suggest that the addition of immunoregulatory mRNA into ETS‐21 loaded with autoantigen mRNA holds the most potential to ameliorate EAE long‐term. In contrast, when the autoantigen was replaced by an irrelevant antigen, the tolerogenic effect was no longer observed, indicating that antigen identity is a critical determinant of the balance between immune tolerance and immune activation. However, the exact mechanism still needs to be elucidated as only modest Treg induction could be observed.

## 3 Discussion

A major unmet need in autoimmune disease therapy is the ability to re‐establish antigen‐specific immune tolerance without evoking generalized immunosuppression. While recent clinical studies in systemic lupus erythematosus and myasthenia gravis [12, 13, 33] demonstrate that CAR T cell‐mediated B cell depletion can durably reset immune dysregulation, the cost and complexity of cellular therapies severely limit their scalability. Tolerogenic vaccines capable of restoring immune homeostasis in an antigen‐ and disease‐specific manner therefore represent a compelling and potentially transformative alternative, particularly if applied early in disease. Here, we demonstrate that systemically and intramuscularly administered mRNA‐LNP vaccines encoding autoantigens robustly suppress disease in two preclinical models of autoimmunity. These findings add to an increasing number of reports that establish mRNA‐LNP technology as a viable therapeutic platform for tolerance induction, rather than solely immune activation [5, 34, 35]. Importantly, our data challenge the prevailing paradigm that tolerogenic mRNA vaccines require the design of fully immune‐silent LNPs and ionizable lipids [10, 14]. Instead, we show that partial, controlled maturation of APCs by LNPs can support, rather than impede, the induction of antigen‐specific tolerance. Moreover, we outline a strategy to further enhance efficacy and durability by co‐delivering mRNA payloads encoding Treg‐promoting factors.

mRNA platforms are uniquely suited for vaccine applications due to their rapid design, modularity, and capacity for multiplexed payload delivery. Conventional i.m. administration with mRNA‐LNPs is thought to strongly favor effector immune responses, driven by the inherent immunostimulatory properties of both the nature of the mRNA and its LNP carrier [16, 17, 36]. Peripheral tolerance, by contrast, is considered to require antigen presentation of an autoantigen under conditions of limited co‐stimulation and inflammation [37, 38]. In this study, we demonstrate that autoantigen mRNA‐LNP vaccines provide efficacy in preclinical models of autoimmunity using both i.v. and i.m. routes of administration, even when using immunostimulatory LNPs, showing that they can be repurposed from prophylactic immune activation to therapeutic immune suppression.

Using a low‐inflammatory, biodegradable ionizable lipid (ETG‐23 [18]) to deliver autoantigen‐encoding mRNA, we achieved marked therapeutic efficacy in murine models of MS and T1D. Notably, in the EAE model, MOG mRNA‐LNP vaccination associates with improved outcome compared with the MS‐approved drug fingolimod under the specific experimental conditions, resulting in faster and sustained disease suppression. Although orally administered in the clinic, i.p. administration of fingolimod is often used as a control for systemic delivery [39]. As an S1PR modulator, lymphocytes are prevented from migrating out of lymphoid organs. Treatment with MOG mRNA‐LNPs led to a pronounced reduction in IFN‐γ^+^ and IL‐17^+^ T cells in the CNS without this cell retention. In addition, in contrast to fingolimod, which required continuous administration to maintain efficacy, the mRNA‐LNP approach conferred durable benefit after only two doses, highlighting its translational potential. Surprisingly, ETS‐21, a structurally related ionizable lipid previously shown to evoke strong effector T cell and B cell responses when delivering microbial antigens i.m. [15], provided even greater therapeutic efficacy upon systemic and i.m. administration in the EAE model. Furthermore, delivery of MOG mRNA by SM‐102, the ionizable lipid used in Moderna's COVID‐19 vaccines [19], also significantly ameliorated EAE disease. This finding challenges the notion that adjuvanticity and immunogenicity are intrinsically linked to ionizable lipid chemistry and LNP composition, yet instead indicates that the immunological outcome of mRNA‐LNP administration is heavily influenced by the nature of the delivered antigen. While these formulations promote immune activation when delivering foreign antigens in vaccination settings, delivery of an autoantigen in the context of autoimmunity appears to induce antigen‐specific tolerance. This divergent outcome can be explained by fundamental differences in T cell responses to self‐ versus foreign antigens [40]. Our APC and T cell data are consistent with this concept, as delivery of autoantigen mRNA by LNPs promoted a mature yet homeostatic APC phenotype, which was accompanied by the induction of hyporesponsive CD4^+^ T cells. Furthermore, the observation that IL‐2 or CCL1 mRNA aggravated disease in the absence of the autoantigen supports the idea that antigen identity is a key determinant of therapeutic outcome, influencing whether immune modulation results in tolerance or pathogenic immune activation.

To our knowledge, this study represents one of the first showing that intramuscular administration of antigen‐encoding mRNA‐LNPs can effectively modulate disease in an autoimmune setting. This finding is further supported by recent work from Lee et al. [41], who reported therapeutic efficacy of intramuscularly administered mRNA‐LNPs in a mouse model of systemic sclerosis. Together, these studies highlight the potential of intramuscular delivery as a clinically beneficial route for mRNA‐based immunotherapies. Importantly, the therapeutic efficacy observed following intramuscular administration may alleviate some of the concerns associated with systemic delivery while leveraging a route of administration with an established safety record from widespread use of mRNA‐LNP vaccines. The ability to achieve disease modulation through a practical and clinically accepted route may therefore facilitate future translation of this therapeutic strategy.

Mechanistically, tolerance induction involves multiple layers of immune modulation. Systemic injected LNPs efficiently delivered mRNA cargo to the liver and spleen, targeting diverse APC populations, including LSECs, Kupffer cells, splenic macrophages, B cells, and DCs. Flow cytometric analyses revealed increased frequencies of mature, CCR7‐expressing DCs in the spleen, yet only at 16 hpi and not at 2 hpi. In parallel, expression of co‐stimulatory markers CD80 and CD86, recently labeled immunogenic molecule PD‐L1 [28], and homeostatic marker ICOS‐L was moderately increased relative to TBS, with more pronounced effects observed for ETS‐21. Crucially, in comparison to the potent TLR3 agonist polyI:C, upregulation of co‐stimulatory markers CD80 and CD86 by i.v. delivery of ETS‐21 loaded with MOG mRNA remained limited, suggesting that in response to mRNA‐LNP exposure, APCs adopt a homeostatic, rather than an immunogenic, phenotype. This phenotype closely resembles homeostatic APC maturation, which has been implicated in the maintenance of peripheral tolerance [26, 42].

Autoantigen mRNA‐LNPs did drive the initial proliferation of MOG‐specific T cells but directed this antigen‐specific response toward apoptosis or to an exhausted and anti‐inflammatory phenotype, rather than toward a pathogenic T cell response. No elevation of MOG‐specific FOXP3^+^ T cells was, however, observed after mRNA‐LNP treatment, arguing against a significant expansion of Tregs by systemic vaccination with autoantigen mRNA‐LNPs. These findings are in line with a recent study that used ALC‐0315 LNPs to deliver an ovalbumin (OVA) peptide i.v., which induced proliferation of OVA‐specific CD4^+^ T cells without promoting FOXP3^+^ Treg differentiation [28]. However, we show increased IL‐10 expression by antigen‐specific cells, which might indicate induction of type 1 regulatory T (Tr1) cells [43]. However, additional markers, such as LAG‐3 and CD49b, need to be investigated to identify Tr1 cells.

While the acute therapeutic benefits of antigen‐specific mRNA‐LNP were demonstrated, our finding that prophylactic immunization with ETS‐21‐MOG could only mildly protect against subsequent EAE induction suggests a transient therapeutic effect. This short duration is likely due to a lack of robust induction of stable Tregs, which are needed for providing durable tolerance. To remedy this shortcoming, we decided to exploit the versatility of mRNA technologies by co‐encapsulating mRNAs encoding factors that stimulate Treg expansion and/or recruitment alongside the autoantigen mRNA. Two such factors were evaluated. IL‐2 muteins, engineered IL‐2 variants with increased selectivity for Tregs, have been shown to expand FOXP3^+^ Tregs in vivo. Repeated dosing with an IL‐2 mutein mRNA‐LNP resulted in generalized, non‐antigen‐specific Treg expansion and delayed disease onset in the EAE model [30, 31, 44]. As a second strategy, we evaluated CCL1, a chemokine implicated in the recruitment and expansion of CCR8^+^ Tregs [32, 45]. We hypothesized that co‐delivery of these factors with autoantigen mRNA would preferentially promote antigen‐specific Treg responses. While co‐delivery of IL‐2 mutein mRNA only modestly delayed disease onset without preventing full disease development, inclusion of CCL1 had a more pronounced protective effect, suppressing disease progression and leaving several animals disease‐free.

Surprisingly, the observed efficacy in this prophylactic setting could not be directly linked to increased frequencies of splenic Tregs at the assessed time points. This suggests that alternative mechanisms, such as localized Treg activity, altered trafficking, or effects on non‐Treg populations, may contribute to disease protection. In addition, while our data demonstrate proof‐of‐concept that homeostatic APC maturation can support tolerance induction, they do not establish generalizable design rules for all mRNA‐LNP formulations or disease contexts. Systematic evaluation of lipid chemistry, dosing regimens, and antigen selection will be required to optimize safety and efficacy across different autoimmune indications.

Altogether, further studies are required to elucidate the precise mechanisms of action and to define optimal combinations, dosing, and timing of autoantigen and immunoregulatory mRNA payloads. Whether this approach can induce bystander tolerance, a critical consideration given antigen heterogeneity in human autoimmune disease, remains an important open question.

## 4 Conclusion

Our findings position systemically and intramuscularly delivered autoantigen mRNA‐LNP vaccines as a scalable, modular, and potentially durable therapeutic strategy for antigen‐specific immune tolerance in autoimmune disease. Collectively, our results redefine design principles for tolerogenic mRNA‐LNP vaccines. Rather than striving to engineer completely innate‐silent LNPs, our data suggest that effective tolerance induction may require a precisely tuned degree of APC activation that enables antigen presentation in a regulatory, non‐inflammatory, autoantigen context. Furthermore, co‐delivery of mRNAs encoding immunoregulatory factors emerges as a promising strategy to enhance both the magnitude and durability of tolerance induction. Prior to clinical translation, additional work will be required to identify optimal mRNA payload combinations and to define which APC populations are most critical for establishing long‐lasting immune tolerance.

Finally, long‐term safety, including the potential impact of repeated systemic mRNA‐LNP administration on innate immune activation, tissue inflammation, or off‐target immune suppression, still needs to be assessed. Reassuringly, repeated intramuscular administration of mRNA‐LNPs has demonstrated a favorable safety profile in vaccination settings. The comparable therapeutic efficacy observed following systemic and intramuscular administration in this study further supports the use of the less invasive intramuscular route, which may offer important advantages for clinical implementation. These considerations will be particularly important for chronic autoimmune diseases that may require repeated or sustained therapeutic intervention.

## 5 Methods

### 5.1 mRNA Development

IL‐2 wildtype (WT) or IL‐2 mutein mRNA encoded either the mus musculus IL‐2 WT or a mutant (N103R‐V106D) fused to a murine serum albumin (MSA) (Table S1). Likewise, CCL1 WT or CCL1 MSA encoded the mus musculus WT CCL1 alone or fused to MSA. MOG mRNA encoded the WT MOG_27‐63_ fused to a DC‐Lamp (Figure S1). All mRNAs were prepared in vitro by T7‐mediated transcription from linearized DNA templates (peTheRNAvs3 vector), which incorporates 5′ and 3′ UTRs and a polyA tail. The final mRNA utilizes Cap1 and 100% replacement of uridine with N1‐Methylpseudouridine (N1*𝜓*), and double‐stranded RNA was removed using silica and cellulose purification methodologies.

### 5.2 LNP Development

All reagents were purchased from commercial sources and used without further purification unless specified otherwise. DSPC and DMGPEG2000 were purchased from Avanti Polar Lipids. Lipid nanoparticles (LNPs, Figure S16) were produced by mixing of an mRNA solution in sodium acetate buffer (100 mM, pH 4) and a lipid solution in ethanol at an aqueous/organic phase flow rate ratio of 3:1 using a T‐mixing set‐up. A nitrogen/phosphate (N/P) ratio of 10 (ETG‐23) or 6 (ETS‐21; MC3) and a total flow rate (TFR) between 12 and 16 mL/min was used, depending on which ionizable lipid was used. Benchmark LNPs were manufactured under similar conditions using parameters as stated in the literature. The lipid solution contained a mixture of ETG‐23 or ETS‐21 ionizable lipid (IL), phospholipid (PL) 1,2‐distearoyl‐sn‐glycero‐3‐phosphocholine (DSPC) (Avanti Polar Lipids), Cholesterol (Chol) (Avanti Polar Lipids), and DMG‐PEG2000 (Avanti Polar Lipids), dissolved in absolute ethanol at a final lipid concentration of 13.33 mM. For ETG‐23, the 4 lipids were mixed at a fixed molar ratio that contains 47,5 mol.% ionizable lipid, 10,5 mol.% phospholipid, 40,5 mol.% cholesterol, and 1.5 mol.% DMG‐PEG2000. For ETS‐21, the 4 lipids were mixed at a fixed molar ratio that contains 50 mol.% ionizable lipid, 10 mol.% phospholipid, 38.5 mol.% cholesterol, and 1.5 mol.% DMG‐PEG2000. After microfluidic mixing of aqueous and organic fraction, LNPs were dialyzed against Tris‐buffered saline (TBS) (20 mM Tris, 0.9% NaCl, pH 8) (10 000 times more TBS volume than LNP volume) using slide‐a‐lyzer dialysis cassettes (20 K Molecular weight cut‐off (MWCO), 3 mL, ThermoFisher). Amicon Ultra Centrifugal Filters (Merck Millipore, 100 kDa MWCO) were used for concentration of LNPs to the desired final concentration in TBS (i.e., 100 µg/mL). LNPs dialyzed against TBS were stored at 2–8°C. Size and polydispersity index were measured with a Zetasizer Nano ZS (Malvern Instruments Ltd., Malvern, U.K.). mRNA encapsulation efficiency was determined via Quant‐iT Ribogreen RNA assay (Thermo Fisher). A range of physicochemical properties is listed in Table S2 for each ionizable lipid used.

### 5.3 In Vitro Assays

A549‐Dual reporter (Invivogen) cells were transfected with mRNA and evaluated at 24 h post‐transfection for interferon response factor activation. mRNA samples were complexed with Lipofectamine MessengerMax (Thermo Fisher Scientific) reagent in a 1 µg mRNA: 3 µL MM ratio per sample (100 ng/well). The complex was incubated for 5 min, and 20 µL was added to a flat‐bottom 96‐well plate. As a positive control, 100 ng/mL Poly IC (high MW) was complexed with Lipofectamine MessengerMax and added to the relevant wells at 20 ng/well. To this plate, 50 000 cells/well in 180 µL complete medium were seeded on top of the MM‐RNA complex and placed in an incubator at 37°C and 5% CO_2_.

### 5.4 Animals and Treatment

All experiments and care of the animals were in accordance with ARRIVE guidelines and institutional guidelines. For all experiments, mice were randomized, and researchers were blinded. In accordance with institutional animal welfare guidelines, mice were monitored regularly for changes in body weight throughout the experiments. In general, animals were not permitted to exceed a weight loss of 15% of their maximal body weight. In specific experimental protocols, for example, EAE, weight loss of up to 25% was permitted for TBS‐treated animals when scientifically justified and explicitly approved by the local animal ethics committee as part of the respective in vivo study protocol, since the manufacturer's protocol mentioned weight loss of approximately 20% during disease progression. A series of independent in vivo experiments were conducted.

For the T1D experiment, female NOD.Cg‐Tg (TcraBDC2.5,TcrbBDC2.5)1Doi/DoiJ (BDC2.5 mice) and NOD.Cg‐Prkdc^scid^/J (NOD‐SCID) mice were purchased from the Jackson Laboratory at 6 weeks of age. All animals were housed in a specific pathogen‐free facility at UMass Chan Medical School, in microisolator cages, given autoclaved food, and maintained on acidified autoclaved water on alternating weeks. All animal use was in accordance with the guidelines of the Animal Care and Use Committee of the UMass Chan Medical School (IACUC approval number: IPROTO202300000080) and conformed to the recommendations in the Guide for the Care and Use of Laboratory Animals (Institute of Laboratory Animal Resources, National Research Council, National Academy of Sciences, 1996). Mice were i.v. treated at indicated timepoints with the indicated LNPs loaded with mRNA constructs (5 µg/dose), or TBS.

For the EAE, 2D2 transfer, and FOXP3 transgenic mice experiments, animals were housed in an accredited conventional animal facility under a 12 h light/dark cycle and had free access to food and water. All mouse procedures were approved by the Hasselt University Ethics Committee for Animal Experiments (local approval numbers: 202334, 202355, 202452, 202454, 202480, 202503). Mice were i.v., i.m., or i.p. treated at indicated timepoints with different LNPs loaded with different mRNA constructs (5‐20 µg/dose), TBS, or fingolimod. For this procedure, mice were anesthetized using 3% isoflurane.

For other in vivo experiments, mice were housed in individually ventilated cages (IVC) under specific pathogen‐free conditions. All animal experiments were performed with approval from the Ethical Committee for Animal Experiments of the Faculty of Medicine and Health Sciences of Ghent University (local approval number: ECD24/71) and from the Ethical Committee for Animal Experiments of the Faculty of Science of VIB at Ghent University (local approval number: EC2024‐051), and animal care was according to established guidelines. These procedures followed the guidelines of the Belgian Council for Laboratory Animal Science (BCLAS), based on policies of the Federation of European Laboratory Animal Science Associations (FELASA) and EU directive 2010/63/EU.

### 5.5 Toxicity Study

Mice were injected i.v. via the tail vein with 60 µg of LNP‐formulated mRNA coding for MOG. Blood collected at 24 h post‐injection was used to check toxicity markers in serum. Briefly, blood was collected from mice through submandibular bleeding and transferred into tubes containing Serum Gel CAT to prevent clotting (Sarstedt, 41.1500.005). Blood was centrifuged at 10 000 × g for 10 min, and serum was collected, transferred to IDEXX tubes, and stored at −80°C until sending to IDEXX. Samples were sent frozen and tested for toxicity markers by IDEXX. A minimum of 100 µL of serum was needed. A standard toxicity panel was tested, including: Alanine Aminotransferase (ALT), Aspartate Aminotransferase (AST), Blood Urea Nitrogen (BUN), BUN:Creatinine Ratio, Creatine Kinase (CK), Creatinine (CREA), Obtained results were sent to etherna and used for analysis.

### 5.6 EAE Model

Ten‐week‐old female C57BL/6 mice were subcutaneously injected with MOG_35‐55_ emulsified in complete Freund's adjuvant containing Mycobacterium tuberculosis, according to the manufacturer's instructions (Hooke Laboratories). Immediately after immunization and on day 1, mice were i.p. injected with 100 ng/100 µL pertussis toxin (PTX). Animals were weighed daily, and neurological deficits were evaluated using a standard five‐point scale (0: no symptoms; 1: limp tail; 2: weakness of hind legs; 3: complete paralysis of hind legs; 4: complete hind and partial front leg paralysis; 5: death). When indicated, spleen, spinal cord, and brain were isolated after transcardial perfusion with Ringer's solution. A single cell suspension from the spleen was derived by mechanical transfer through a 70 µm cell strainer (Greiner Bio‐One). For the CNS, both enzymatic digestion, using collagenase D (Roche Diagnostics GmbH) and DNase I (Roche Diagnostics GmbH), and mechanical dissociation was performed, followed by a Percoll gradient (GE Healthcare). Isolated cells were further investigated using flow cytometry. Isolated cells were stimulated with phorbol 12‐myristate 13‐acetate (PMA, Merck), ionomycin (CaI, Merck), and Golgiplug (BD Biosciences) for 4 h at 37°C at 5% CO_2_. Next, cells were blocked with CD16/32 (93, BioLegend) and stained with Zombie Aqua, CD3 BV785 (17A2), CD4 APC‐Fire750 (RM4‐5), and CD25 PE (PC61; all BioLegend). After fixation and permeabilization using FOXP3 transcription factor staining kit (Invitrogen), cells were stained with IFN‐γ BV605 (XMG1.2) and IL‐17 BV421 (TC11‐18H10.1; all BioLegend) and were acquired on BD LSRFortessa (BD Biosciences) and analyzed using FlowJo 10.10.0 (BD Biosciences). Cell counts were calculated by multiplying the number of cells of an organ counted based on size with the percentage of a certain subset within the size gate used for counting.

### 5.7 T1D Model

BDC2.5 mice were used as a source for autoreactive diabetogenic CD4 T cells. Splenocytes from BDC2.5 mice were cultured for 2 days in RPMI 1640 medium (Life Technologies) supplemented with 10% fetal bovine serum, 100 U/mL penicillin G, 100 µg/mL streptomycin sulfate, and 2 mM l‐glutamine in the presence of p31 peptide (1 µM). Total CD4^+^ T cells were purified by negative selection using MACS columns. NOD‐SCID recipients were injected i.v. with 2 × 10^6^ BDC2.5 CD4 T cells. Blood glucose levels were measured twice a week using an Accuchek Aviva Plus glucometer (Hoffman‐La Roche Ltd) to confirm diabetes (blood glucose > 250 mg/dL). At day 8, cells were recovered from the blood, spleen, or pancreas and evaluated for levels of mouse CD4 T cells. At 30 days of induction, CD4 T cells were measured in the blood and assessed for expression of PD‐1. Next, cells were blocked with CD16/32 (93, BioLegend) and stained with Live/Dead Near IR (ThermoFisher), CD45 PerCP‐Cy5.5 (30‐F11), CD3 BV421 (17A2), CD4 FITC (RM4‐5), CD8 APC (53‐5.8), and PD‐1 PE (29F.1A12) from all BioLegend. Cells were analyzed using a BD FACSymphony A5 Cell Analyzer, and flow cytometry data were analyzed with FlowJo (TreeStar). Pancreatic tissues were collected from mice at the indicated time points, and the specimens were fixed in formalin and embedded in paraffin for immunohistochemistry analyses. Hematoxylin and Eosin (H&E) stains were performed on formalin‐fixed, paraffin‐embedded mouse pancreatic tissue blocks. Insulitis was scored histologically on a scale of 0–4 on the basis of the extent of immune cell infiltration and islet destruction. A score of 0 indicated intact islets without lymphocytic infiltration. A score of 1 indicated peri‐insulitis, defined as immune cells confined to the islet periphery without invasion of the islet parenchyma. A score of 2 indicated invasive insulitis involving < 50% of the islet area. A score of 3 indicated severe insulitis involving ≥ 50% of the islet area. A score of 4 indicated end‐stage insulitis, defined by collapse of islet architecture with minimal or no remaining intact islet mass. Scoring was done blindly, and a minimum of 15 islets were scored per mouse. For insulin immunofluorescence staining, selected formalin‐fixed, paraffin‐embedded mouse pancreatic tissue blocks were sectioned at 5 µm and mounted on glass slides. Slides were deparaffinized in xylene and rehydrated. Slides were blocked with 2.5% horse serum (Vector Laboratories, MP‐7401) for 20 min. Primary antibody incubation was performed for 30 min at RT using anti‐insulin antibody (Abcam, ab181547; 1:2000 dilution) diluted in SignalStain antibody diluent (Cell Signaling Technology, 8112L). Control sections were incubated with rabbit IgG (Thermo Fisher Scientific, NI01; 1:188 dilution) in place of the primary antibody. Slides were washed and incubated with horse anti‐rabbit HRP secondary reagent (Vector Laboratories, MP‐7401) for 30 min at RT. The immunofluorescence signal was developed using Vector tyramide signal amplification (TSA) with AZDye 555 reagent (Vector Laboratories, CCT‐1542) for 10 min at RT. The TSA working solution was prepared at a 1:1:100 ratio of AZDye 555 reagent, 0.15% hydrogen peroxide, and PBS. Slides were then washed, counterstained with DAPI (1:500 in PBS) for 5 min at RT, washed, and then cover‐slipped. Samples were stained, and images were acquired by Applied Pathology Systems.

### 5.8 In Vivo Imaging System (IVIS)

For in vivo imaging, mice were injected i.v. via the tail vein with 5 µg (25 µg/mL) of LNP‐formulated mRNA coding for Fluc. To monitor Fluc activity, 100 µL of 30 mg/mL D‐luciferin (E1605, Promega) in 0.9% Potassium salt solution was injected i.p., 24 h after mRNA/LNP injection. Images were taken 10–15 min after injection of luciferin to reach the emission peak. The mice were sedated and monitored using an IVIS Lumina S5 imaging system. Photon flux was quantified using the Living Image software (all from Caliper Life Sciences). Regions of interest (ROIs) were quantified as average radiance (photons/[s cm^2^ sr]) or total flux (photons/s) represented as color‐scaled images superimposed on grayscale photos of mice using Living Image software (Caliper Life Sciences).

### 5.9 Cellular Tropism

Female 7‐week‐old BALB/c mice were purchased from Charles River. Mice were injected i.v. with 20 µg of LNP containing CD90.1 mRNA or with TBS as a control. 24 h later, mice were euthanized, and the liver and spleen were collected for flow cytometry. Liver and spleen were isolated and collected in MACS storage buffer tissue. Liver was dissociated with a liver dissociation Kit (Miltenyi), following the manufacturer's protocol, and further processed with a gentle MACS Dissociator with Heaters run “m_liver_03” program (Miltenyi), followed by 30 min incubation at 37°C under continuous rotation using the SW22 rotating bath (Analis) and a final run with the gentleMACS Program “m_liver_04” (Miltenyi). Spleen was digested using spleen dissociation kit and GentleMACS Dissociator with Heaters (Miltenyi Biotec) per manufacturer's instructions, and further processed with gentle MACS Dissociator with Heaters run “m_spleen_02_01” program (Miltenyi), followed by 15 min incubation at 37°C under continuous rotation using the SW22 rotating bath (Analis) and a final run with the gentleMACS Program “m_spleen_03_02” (Miltenyi). After obtaining a cell suspension from liver or spleen tissue, red blood cell lysis was performed (5 min, 4°C). Cell number was evaluated using a Cellaca MX automated cell counter (Revvity).

### 5.10 Assessment of Serum Cytokines/Chemokines

Blood collected at 5 h post‐injection was used to check for inflammatory cytokines/chemokines by means of Legendplex (Biolegend) with a custom‐made pre‐mixed cytokine panel (900001482). The cytokines/chemokines included were CCL2 (MCP‐1), CXCL10 (IP‐10), IFN‐α, IFN‐γ‐12p70, IL‐6, and TNF‐α. 12.5 µL of each serum sample test was added to the V‐bottom plate (Biolegend), and 12.5 µL Assay Buffer was added. After vortexing the pre‐mixed beads for 1 min, 12.5 µL of the bead suspension was added to each well on the plate. The plate was sealed and wrapped in aluminum foil (VWR) to protect from light and incubated with vigorous shaking (800 rpm) for 2 h at RT using a plate shaker (Titramax 101, Heidolph). After this, the plates were centrifuged at 250 g for 5 min at RT in a tabletop centrifuge with plate adaptors (Eppendorf). The plates were decisively inverted over a sink and tapped onto a piece of absorbing paper to blot excess remaining fluid. The beads were washed by resuspending in 200 µL wash buffer, and a second centrifugation plus decanting event followed. An incubation step of 1 h with 12.5 µL detection Ab per well was performed, in sealed plates, wrapped in aluminum foil, shaking at 800 rpm. The Streptavidin‐PE reagent was added, 12.5 µL per well, and the plates were incubated shaking for another 30 min.  After the streptavidin incubation, another wash/decant step was performed, and the beads were resuspended in 150 µL Wash buffer, and fluorescence was measured by Flow Cytometry (Cytoflex LX, Beckman & Coulter) using the APC channel (for bead populations) and the PE channel (antibody binding intensity) with a pre‐defined template for bead population gating. The data was collected and analyzed using the Legendplex Qognit tool (https://legendplex.qognit.com/.

Samples for flow cytometry analysis were incubated with Live/dead Fixable Near IR (876) Viability (ThermoFisher) and anti‐CD16/CD32 (clone 2.4G2), to prevent aspecific binding, for 15 min at RT. Cell suspensions (2 × 10^6^ cells/well) were then incubated for 30 min at 4°C with fluorescently labeled antibodies diluted in FACS buffer (PBS supplemented with 1% BSA and 0.09% sodium azide) and 1/10 BD Horizon Brilliant Stain Buffer. After incubation, wells were washed with FACS buffer and centrifugated at 300 g for 3 min. Flow cytometry data were acquired using a Cytoflex LX (Beckman Coulter) and analyzed using FlowJo. A list of antibodies used can be found in Table S3.

### 5.11 DC Phenotyping

Female C57/BL6J mice were injected i.v. via the tail vein with 5 µg of LNP‐formulated mRNA coding for MOG mRNA. 2 h or 16 h later, spleens were minced manually with scissors before digestion in RPMI 1640 (Thermo Fisher Scientific, 21875‐059) containing Liberase TM (0.02 mg/mL; Roche, 05 401 127 001) and recombinant DNase I (10U/mL; Roche, 04 536 282 001) for 30 min at 37°C. Red blood cells were removed by osmotic lysis (homemade buffer), followed by passage through a 70 mm cell strainer (Falcon, 734‐0003). Prior to antibody staining, live cells were counted by staining with acridine orange/propidium iodide (Logos Biosystems, LB F23001) and using the LUNA‐FX7 (Logos Biosystems). 4–5 × 10^6^ cells were stained by several antibody staining steps with fluorochrome‐ or biotin‐conjugated antibodies. An initial staining step consisted of Fc block (Polpharma Biologics), CD64‐BV711 (BioLegend), and biotinylated ICOSL antibody (BioLegend) for 45 min at 4°C. A second staining step included all other antibodies, including streptavidin‐PE‐CF594 (BD Biosciences, 562284), to stain the remaining surface proteins with incubation of 30 min at 4°C. Viability was assessed by use of Fixable Viability Dye eFluor 506 (Thermo Fisher Scientific, 65‐0866‐14). Flow cytometry was performed on FACSymphony A5 using FACSDiva software (BD Biosciences). To adjust photomultiplier tube voltages and to calculate the compensation matrix, single‐stained cells and UltraComp eBeads (Thermo Fisher Scientific, 01‐2222‐42) were used. A list of antibodies used can be found in Table S4.

### 5.12 In Vivo Expression

Mice were i.v. injected with LNPs loaded with different mRNA constructs (5–20 µg/dose) and blood, liver, or spleen were isolated from mice at the indicated timepoints. Mice were bled submandibular, and blood was collected in micro sample tubes containing a serum Gel that prevents clotting (Sarstedt, 41.1500.005). Blood was centrifuged at 10 000 × g for 10 min, and serum was collected. Serum was frozen at ‐20°C until use. CCL1 and IL‐2 levels were quantified using the mouse TCA‐3/CCL1 ELISA Kit (Invitrogen, EMCCL1) and mouse IL‐2 Duoset ELISA (R&D Systems, DY402) according to the manufacturer's instructions. Absorbance was measured with the Tecan Infinite 200pro Mplex. Isolated immune cells were analyzed using flow cytometry as described below.

### 5.13 In Vivo Treg Expansion Assay

FOXP3 transgenic mice, BAC‐FOXP3Cre‐GFP mice, and Rosa26flSTOPflRFP (FOXP3 reporter) mice, with a C57BL/6 background, were crossed. This model allows identification of FOXP3^+^GFP^+^RFP^+^ Tregs and discrimination from GFP^−^RFP^+^ exFOXP3 Tregs. 12‐week‐old mice of both sexes were once i.v. injected with 5 µg/dose of mRNA encapsulated in LNP or TBS as a TBS. Four days after injection, the spleen was harvested, and a single‐cell suspension was derived by mechanical transfer through a 70 µm cell strainer. Cells were treated with 0.83% ammonium chloride for 4 min. Next, immune cells were further investigated with flow cytometry. Cells were blocked with CD16/32 (93, BioLegend) and stained with Zombie Aqua, CD3 BV785 (17A2), CD4 BV605 (RM4‐5), and CD25 APC (PC61; all BioLegend). Cells were acquired on BD LSRFortessa (BD Biosciences) and analysed using FlowJo 10.10.0 (BD Biosciences). Cell counts were calculated by multiplying the number of cells of an organ counted based on size by the percentage of a certain subset within the size gate used for counting.

### 5.14 2D2 model

The spleens were isolated from at least 6‐week‐old female 2D2 mice (C57BL/6‐Tg(Tcra2D2,Tcrb2D2)1Kuch/J [29]). A single cell suspension from the spleen was derived by mechanical transfer through a 70 µm cell strainer (Greiner Bio‐One). CD4 T cells were MACS‐sorted using the CD4 T cell isolation kit (Miltenyi Biotec). When needed, CD4 T cells were labelled with CellTrace Violet (5 µM, Thermo Fisher Scientific) prior to i.v. injection (1 million cells in 200 µL TBS) into 8–10 week old female, CD90.1 (CD90.2^−^) recipient mice (B6.PL‐Thy1a/CyJ). When indicated, recipient mice were actively induced with MOG_35‐55_ EAE as described before. At the specified timepoints, spleens were isolated and single‐cell suspensions were derived as described previously. When indicated, CD4 T cells were MACS‐sorted. Isolated cells were further investigated using flow cytometry. Isolated cells were stimulated with phorbol 12‐myristate 13‐acetate (PMA, Merck), ionomycin (CaI, Merck), and Golgiplug (BD Biosciences) for 4 h at 37°C at 5% CO_2_. Next, cells were blocked with CD16/32 (93, BioLegend) and stained with Zombie NIR, CD3 APC‐Fire810 (17A2), CD4 BV510 (RM4‐5), CD90.2 BV785 (30‐H12), PD‐1 PE‐Fire700 (29F.1A12), Apotracker Green, GITR BV711 (DTA‐1), and CD73 BV605 (TY/11.8; all BioLegend). After fixation and permeabilization using FOXP3 transcription factor staining kit (Invitrogen), cells were stained with IL‐10 PE‐Dazzle 594 (JES5‐16E3), FOXP3 PE (150D; all BioLegend), and were acquired on Cytek Aurora (Cytek) and analysed using FlowJo 10.10.0 (BD Biosciences).

### 5.15 Statistical Analysis

Statistical analyses were performed using GraphPad Prism version 10.6.0 (GraphPad Software). Details of statistical tests are given in the figure legends. Outliers were tested using the ROUT's test. Normality was statistically tested, and tests were chosen accordingly. Tests used are: Chi‐square test, Log‐rank (Mantel‐Cox) test, two‐way ANOVA with Tukey's multiple comparisons test, Kruskal‐Wallis with Dunn's multiple comparison test, and Mann‐Whitney test. Cumulative data are shown as mean ± SEM. A p‐value < 0.05 was considered significant. ^*^: *p* < 0.05; ^**^: *p* < 0.01; ^***^: *p* < 0.001; ^****^: *p* < 0.0001.

## Author Contributions

All authors reviewed the manuscript. P.B., K.B., B.B., N.H., F.L., and S.K. conceptualized and designed the research. P.B., T.S., K.B., J.V.D.H., I.V., R.N., J.F., V.M., L.J., and B.V. performed the experiments and analyzed the data. P.B., K.B., B.B., and S.K. prepared the figures and wrote the paper. C.H. designed the mRNA constructs. S.K. and E.S were involved in the formulation of lipid nanoparticles. M.B. designed and executed the type 1 diabetes study. S.J. and S.M. contributed to the phenotyping of the antigen presenting cells. P.B., K.B., and J.D.V. acted as project leader and project manager of the VLAIO project. P.B., T.S., J.V., G.D., D.L., L.S., X.Z., M.S., J.V.B., R.N., B.M., and J.B. assisted in EAE studies.

## Funding

This work was supported by VLAIO (HBC.2023.0802). Studies at UMass were supported by the Barbara D. Cammett Breakthrough T1D Center of Excellence in New England of the Breakthrough T1D Foundation under award number 4‐COE‐2025‐1751‐A‐N.

## Conflicts of Interest

The research is funded by etherna Immunotherapies NV. Karen Beets, Carlo Heirman, Jurgen Van den Heuvel, Bart Vanderborght, Ismael Varela, Roxanne Nouille, Lotte Jacobs, Jessica Filtjens, Sabah Kasmi, Elise Seynaeve, Veronica Mavrovouna, Jana De Vrieze, Florence Lambolez, and Stefaan De Koker are employees of etherna Immunotherapies NV and may hold stock in the company.

## Supporting information




**Supporting File**: advs76382‐sup‐0001‐SuppMat.docx.

## Data Availability

The data that support the findings of this study are available from the corresponding author upon reasonable request.

## References

[1] J. Ludvigsson , D. Krisky , R. Casas , et al., “GAD65 Antigen Therapy in Recently Diagnosed Type 1 Diabetes Mellitus,” New England Journal of Medicine 366, no. 5 (2012): 433–442, 10.1056/NEJMoa1107096. 22296077

[2] B. Bielekova , B. Goodwin , N. Richert , et al., “Encephalitogenic Potential of the Myelin Basic Protein Peptide (amino acids 83–99) in Multiple Sclerosis: Results of a Phase II Clinical Trial with an Altered Peptide Ligand,” Nature Medicine 6, no. 10 (2000): 1167–1175, 10.1038/80516. 11017150

[3] L. Kappos , G. Comi , H. Panitch , et al., “Induction of a Non‐Encephalitogenic Type 2 T Helper‐Cell Autoimmune Response in Multiple Sclerosis after Administration of an Altered Peptide Ligand in a Placebo‐Controlled, Randomized Phase II Trial,” Nature Medicine 6, no. 10 (2000): 1176–1182, 10.1038/80525. 11017151

[4] K. W. Wucherpfennig , A. Sette , S. Southwood , et al., “Structural Requirements for Binding of an Immunodominant Myelin Basic Protein Peptide to DR2 Isotypes and for Its Recognition by Human T Cell Clones,” The Journal of Experimental Medicine 179, no. 1 (1994): 279–290, 10.1084/jem.179.1.279. 7505801 PMC2191316

[5] C. Krienke , L. Kolb , E. Diken , et al., “A Noninflammatory mRNA Vaccine for Treatment of Experimental Autoimmune Encephalomyelitis,” Science 371, no. 6525 (2021): 145–153, 10.1126/science.aay3638. 33414215

[6] M. Gomi , Y. Nakayama , Y. Sakurai , et al., “Tolerogenic Lipid Nanoparticles for Delivering Self‐Antigen mRNA for the Treatment of Experimental Autoimmune Encephalomyelitis,” Pharmaceuticals 16, no. 9 (2023): 1270, 10.3390/ph16091270. 37765078 PMC10537621

[7] S. G. Brew , M. Frey , D. P. McCarthy , A. Elhofy , and R. J. Nowak , “Antigen‐Specific Immune Therapy (CNP‐106) for Treatment of Generalised Myasthenia Gravis: Rationale and Design of First‐in‐Human Randomised Controlled Trial,” BMJ Neurol Open 6, no. 2 (2024): 000836. 10.1136/bmjno-2024-000836 PMC11667273 39720510

[8] R. Wang , L. J. Kubiatowicz , R. Zhang , L. Bao , R. H. Fang , and L. Zhang , “Nanoparticle Approaches for Manipulating Cytokine Delivery and Neutralization,” Frontiers in Immunology 16 (2025): 1592795, 10.3389/fimmu.2025.1592795. 40557148 PMC12185478

[9] D. Didona , C. Hudemann , H. Garn , et al., “Safety, Tolerability, Pharmacokinetics and Pharmacodynamic Effects of Desmoglein 3 Peptide‐coupled Tolerizing Nanoparticles in Pemphigus,” British Journal of Dermatology 194, no. 1 (2026): 86–98, 10.1093/bjd/ljaf311. 40795222

[10] B. E. Nachod , A. S. Thatte , R. Palanki , and M. J. Mitchell , “Nanoparticle‐Based Tolerogenic Vaccines: Next‐Generation Strategies for Autoimmune and Allergic Disease Therapies,” Angewandte Chemie 65 (2025): 24097. 10.1002/anie.202524097 PMC12865243 41451511

[11] F. P. Polack , S. J. Thomas , N. Kitchin , et al., “Safety and Efficacy of the BNT162b2 mRNA Covid‐19 Vaccine,” New England Journal of Medicine 383, no. 27 (2020): 2603–2615, 10.1056/NEJMoa2034577. 33301246 PMC7745181

[12] R. R. Fedak , R. N. Ruggerie , Y. Shan , et al., “BCMA‐directed mRNA CAR‐T Cell Therapy for Myasthenia Gravis: Exploratory Biomarker Analysis of a Placebo‐Controlled Phase 2b Trial,” Nature Medicine 32 (2026): 1118–1130, 10.1038/s41591-025-04170-z. PMC13004674 41514039

[13] A. Mackensen , F. Müller , D. Mougiakakos , et al., “Anti‐CD19 CAR T Cell Therapy for Refractory Systemic Lupus Erythematosus,” Nature Medicine 28, no. 10 (2022): 2124–2132, 10.1038/s41591-022-02017-5. 36109639

[14] R. Razavi , M. Kegel , J. Muscat‐Rivera , D. Weissman , and J. R. Melamed , “Harnessing mRNA‐Lipid Nanoparticles as Innovative Therapies for Autoimmune Diseases,” Molecular Therapy Methods & Clinical Development 33, no. 3 (2025): 101566, 10.1016/j.omtm.2025.101566. 40969676 PMC12441705

[15] R. De Coen , S. Kasmi , S. Dumbre , et al., “Next‐Generation Bio‐Reducible Lipids Enable Enhanced Vaccine Efficacy in Malaria and Primate Models,” Advanced Functional Materials 36 (2025): 09838.

[16] M. G. Alameh , I. Tombácz , E. Bettini , et al., “Lipid Nanoparticles Enhance the Efficacy of mRNA and Protein Subunit Vaccines by Inducing Robust T Follicular Helper Cell and Humoral Responses,” Immunity 54, no. 12 (2021): 2877–2892, 10.1016/j.immuni.2021.11.001. 34852217 PMC8566475

[17] S. Tahtinen , A. J. Tong , P. Himmels , et al., “IL‐1 and IL‐1ra are Key Regulators of the Inflammatory Response to RNA Vaccines,” Nature Immunology 23, no. 4 (2022): 532–542, 10.1038/s41590-022-01160-y. 35332327

[18] E. De Lombaerde , Y. Chen , T. Ye , et al., “Combinatorial Screening of Biscarbamate Ionizable Lipids Identifies a Low Reactogenicity Lipid for Lipid Nanoparticle mRNA Delivery,” Advanced Functional Materials 34, no. 21 (2024): 2310623.

[19] L. Zhang , K. R. More , A. Ojha , et al., “Effect of mRNA‐LNP Components of Two Globally‐Marketed COVID‐19 Vaccines on Efficacy and Stability,” NPJ Vaccines 8, no. 1 (2023): 156, 10.1038/s41541-023-00751-6. 37821446 PMC10567765

[20] A. Bonehill , C. Heirman , S. Tuyaerts , et al., “Messenger RNA‐Electroporated Dendritic Cells Presenting MAGE‐A3 Simultaneously in HLA Class I and Class II Molecules,” The Journal of Immunology 172, no. 11 (2004): 6649–6657, 10.4049/jimmunol.172.11.6649. 15153480

[21] B. De Keersmaecker , C. Heirman , S. Allard , et al., “Lumenal Part of the DC‐LAMP Protein Is Not Required for Induction of Antigen‐Specific T Cell Responses by Means of Antigen‐DC‐LAMP Messenger RNA‐Electroporated Dendritic Cells,” Human Gene Therapy 21, no. 4 (2010): 479–485. 19903083 10.1089/hum.2009.080

[22] W. Li , H. Vanluchene , L. Raes , et al., “Efficacy versus Immunogenicity of LNP‐mediated Delivery of mRNA and Self‐Amplifying RNA Upon Intravitreal Injection in the Mouse Eye,” Journal of Controlled Release 385 (2025): 114027, 10.1016/j.jconrel.2025.114027. 40659060

[23] J. D. Peterson and K. Haskins , “Transfer of Diabetes in the NOD‐ scid Mouse by CD4 T‐Cell Clones: Differential Requirement for CD8 T‐Cells,” Diabetes 45, no. 3 (1996): 328–336, 10.2337/diab.45.3.328. 8593938

[24] A. Akinc , M. A. Maier , M. Manoharan , et al., “The Onpattro Story and the Clinical Translation of Nanomedicines Containing Nucleic Acid‐Based Drugs,” Nature Nanotechnology 14, no. 12 (2019): 1084–1087, 10.1038/s41565-019-0591-y. 31802031

[25] X. Zhang , V. Goel , and G. J. Robbie , “Pharmacokinetics of Patisiran, the First Approved RNA Interference Therapy in Patients with Hereditary Transthyretin‐Mediated Amyloidosis,” The Journal of Clinical Pharmacology 60, no. 5 (2020): 573–585, 10.1002/jcph.1553. 31777097 PMC7187331

[26] V. Bosteels , S. Maréchal , C. De Nolf , et al., “LXR Signaling Controls Homeostatic Dendritic Cell Maturation,” Science Immunology 8, no. 83 (2023): add3955, 10.1126/sciimmunol.add3955. 37172103

[27] M. P. Domogalla , P. V. Rostan , V. K. Raker , and K. Steinbrink , “Tolerance Through Education: How Tolerogenic Dendritic Cells Shape Immunity,” Frontiers in Immunology 8 (2017): 1764, 10.3389/fimmu.2017.01764. 29375543 PMC5770648

[28] S. Rennen , V. Bosteels , C. De Nolf , et al., “Lipid Nanoparticles as a Tool to Dissect Dendritic Cell Maturation Pathways,” Cell Reports 44, no. 8 (2025): 116150, 10.1016/j.celrep.2025.116150. 40828654 PMC12381551

[29] E. Bettelli , M. Pagany , H. L. Weiner , C. Linington , R. A. Sobel , and V. K. Kuchroo , “Myelin Oligodendrocyte Glycoprotein–Specific T Cell Receptor Transgenic Mice Develop Spontaneous Autoimmune Optic Neuritis,” The Journal of Experimental Medicine 197, no. 9 (2003): 1073–1081, 10.1084/jem.20021603. 12732654 PMC2193967

[30] S. de Picciotto , N. DeVita , C. J. Hsiao , et al., “Selective Activation and Expansion of Regulatory T Cells Using Lipid Encapsulated mRNA Encoding a Long‐acting IL‐2 Mutein,” Nature Communications 13, no. 1 (2022): 3866. 10.1038/s41467-022-31130-9 PMC9256694 35790728

[31] L. Khoryati , M. N. Pham , M. Sherve , et al., “An IL‐2 Mutein Engineered to Promote Expansion of Regulatory T Cells Arrests Ongoing Autoimmunity in Mice,” Science Immunology 5, no. 50 (2020): aba5264, 10.1126/sciimmunol.aba5264. PMC7643170 32817295

[32] Y. Barsheshet , G. Wildbaum , E. Levy , et al., “CCR^8+^ FOXp^3+^ T Reg Cells as Master Drivers of Immune Regulation,” Proceedings of the National Academy of Sciences 114, no. 23 (2017): 6086–6091, 10.1073/pnas.1621280114. PMC5468670 28533380

[33] D. Mougiakakos , G. Krönke , S. Völkl , et al., “CD19‐Targeted CAR T Cells in Refractory Systemic Lupus Erythematosus,” New England Journal of Medicine 385, no. 6 (2021): 567–569, 10.1056/NEJMc2107725. 34347960

[34] M. Gomi , Y. Sakurai , M. Sato , et al., “Delivering mRNA to Secondary Lymphoid Tissues by Phosphatidylserine‐Loaded Lipid Nanoparticles,” Advanced Healthcare Materials 12, no. 9 (2023): 2202528, 10.1002/adhm.202202528. 36535635

[35] X. Xu , X. Wang , Y. P. Liao , L. Luo , T. Xia , and A. E. Nel , “Use of a Liver‐Targeting Immune‐Tolerogenic mRNA Lipid Nanoparticle Platform to Treat Peanut‐Induced Anaphylaxis by Single‐ and Multiple‐Epitope Nucleotide Sequence Delivery,” ACS Nano 17, no. 5 (2023): 4942–4957, 10.1021/acsnano.2c12420. 36853930 PMC10019335

[36] N. Chaudhary , L. N. Kasiewicz , A. N. Newby , et al., “Amine Headgroups in Ionizable Lipids Drive Immune Responses to Lipid Nanoparticles by Binding to the Receptors TLR4 and CD1d,” Nature Biomedical Engineering 8, no. 11 (2024): 1483–1498, 10.1038/s41551-024-01256-w. PMC11863198 39363106

[37] D. L. Mueller , “Mechanisms Maintaining Peripheral Tolerance,” Nature Immunology 11, no. 1 (2010): 21–27, 10.1038/ni.1817. 20016506

[38] A. Cifuentes‐Rius , A. Desai , D. Yuen , A. P. R. Johnston , and N. H. Voelcker , “Inducing Immune Tolerance with Dendritic Cell‐targeting Nanomedicines,” Nature Nanotechnology 16, no. 1 (2021): 37–46, 10.1038/s41565-020-00810-2. 33349685

[39] T. Koda , A. Namba , Y. Nakatsuji , et al., “Beneficial Effects of Fingolimod in MS Patients with High Serum Sema4A Levels,” PLoS ONE 13, no. 3 (2018): 0193986, 10.1371/journal.pone.0193986. PMC5843273 29518148

[40] R. M. Teague , P. D. Greenberg , C. Fowler , et al., “Peripheral CD^8+^ T Cell Tolerance to Self‐Proteins is Regulated Proximally at the T Cell Receptor,” Immunity 28, no. 5 (2008): 662–674, 10.1016/j.immuni.2008.03.012. 18424189 PMC3443683

[41] A. R. Lee , S. B. Jeon , H. W. Kwak , et al., “A20 mRNA Therapeutics Ameliorate Systemic Sclerosis by Suppressing TRAF‐6/NF‐KB Signaling and DREAM Expression and Exerting Antifibrotic Effects,” Frontiers in Immunology 16 (2025): 1665998, 10.3389/fimmu.2025.1665998. 41181125 PMC12571734

[42] C. A. Iberg , A. Jones , and D. Hawiger , “Dendritic Cells as Inducers of Peripheral Tolerance,” Trends in Immunology 38, no. 11 (2017): 793–804, 10.1016/j.it.2017.07.007. 28826942 PMC5669994

[43] M. G. Roncarolo , S. Gregori , R. Bacchetta , M. Battaglia , and N. Gagliani , “The Biology of T Regulatory Type 1 Cells and Their Therapeutic Application in Immune‐Mediated Diseases,” Immunity 49, no. 6 (2018): 1004–1019, 10.1016/j.immuni.2018.12.001. 30566879

[44] O. Efe , R. B. Gassen , L. Morena , et al., “A Humanized IL‐2 Mutein Expands Tregs and Prolongs Transplant Survival in Preclinical Models,” Journal of Clinical Investigation 134, no. 5 (2024): 173107, 10.1172/JCI173107. PMC10904054 38426492

[45] R. Wang , J. Xu , S. Cheng , et al., “TNFR2/CCR8 bispecific Antibody Enhances Antitumor Activity through Depleting Ti‐Tregs and Boosting Effector CD^8+^ T Cell Function,” Oncoimmunology 14, no. 1 (2025): 2497171, 10.1080/2162402X.2025.2497171. 40293187 PMC12039408

