# Supplementary material for: Autoantigen mRNA‐LNP Vaccination Drives Therapeutic Efficacy in Preclinical Models for Autoimmunity
Source: Adv Sci (Weinh). 2026 Jul 6:e76382. Online ahead of print. doi: 10.1002/advs.76382 (PMC13335699; doi:10.1002/advs.76382)
Supplement: Supplementary file 1 — Supporting File: advs76382‐sup‐0001‐SuppMat.docx. [file ADVS-9999-e76382-s001.docx]

**Autoantigen mRNA-LNP vaccination drives therapeutic efficacy in preclinical models for autoimmunity**

Paulien Baeten1,2#, Karen Beets3#, Tessa Schalley1,2, Janne Verreycken1,2, Gayel Duran1,2, Daphne Lintsen1,2, Lisa Schuetz1,2,4, Xue Zhong1,2, Rinke Nieuwschepen1,2, Melissa Schepers1,5,6, Brecht Moonen1,2, Jeroen Bogie1,2, Jana Van Broeckhoven1,2, Carlo Heirman3, Jurgen Van den Heuvel3, Bart Vanderborght3, Ismael Varela3, Roxanne Nouille3, Lotte Jacobs3, Jessica Filtjens3, Sabah Kasmi3, Elise Seynaeve3, Veronica Mavrovouna3, Jana De Vrieze3, Michael A. Brehm7, Sandra Maréchal8, Sophie Janssens8, Florence Lambolez3, Niels Hellings1,2, Stefaan De Koker3#, Bieke Broux1,2#*

**Supplementary Figures and Tables**


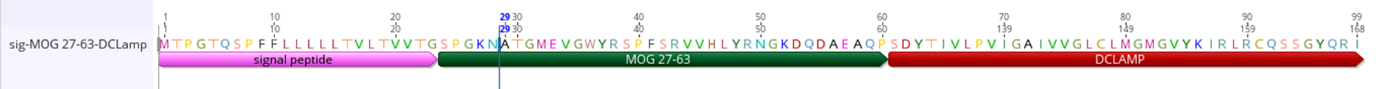


**Figure S1. mRNA construct design of MOG mRNA**


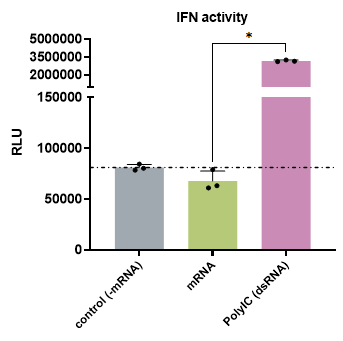


**Figure S2. *In vitro* transfection of mRNA in A549-Dual reporter cells shows no elevations in IFN activity.** A549-Dual reporter cells were transfected with mRNA (100ng/well), buffer control or polyI:C as positive control (100ng/ml). Innate activation was assessed through interferon (IFN) activity 24 h post transfection. n=3; Kruskal-Wallis test. *: p<0.05. RLU: relative light units


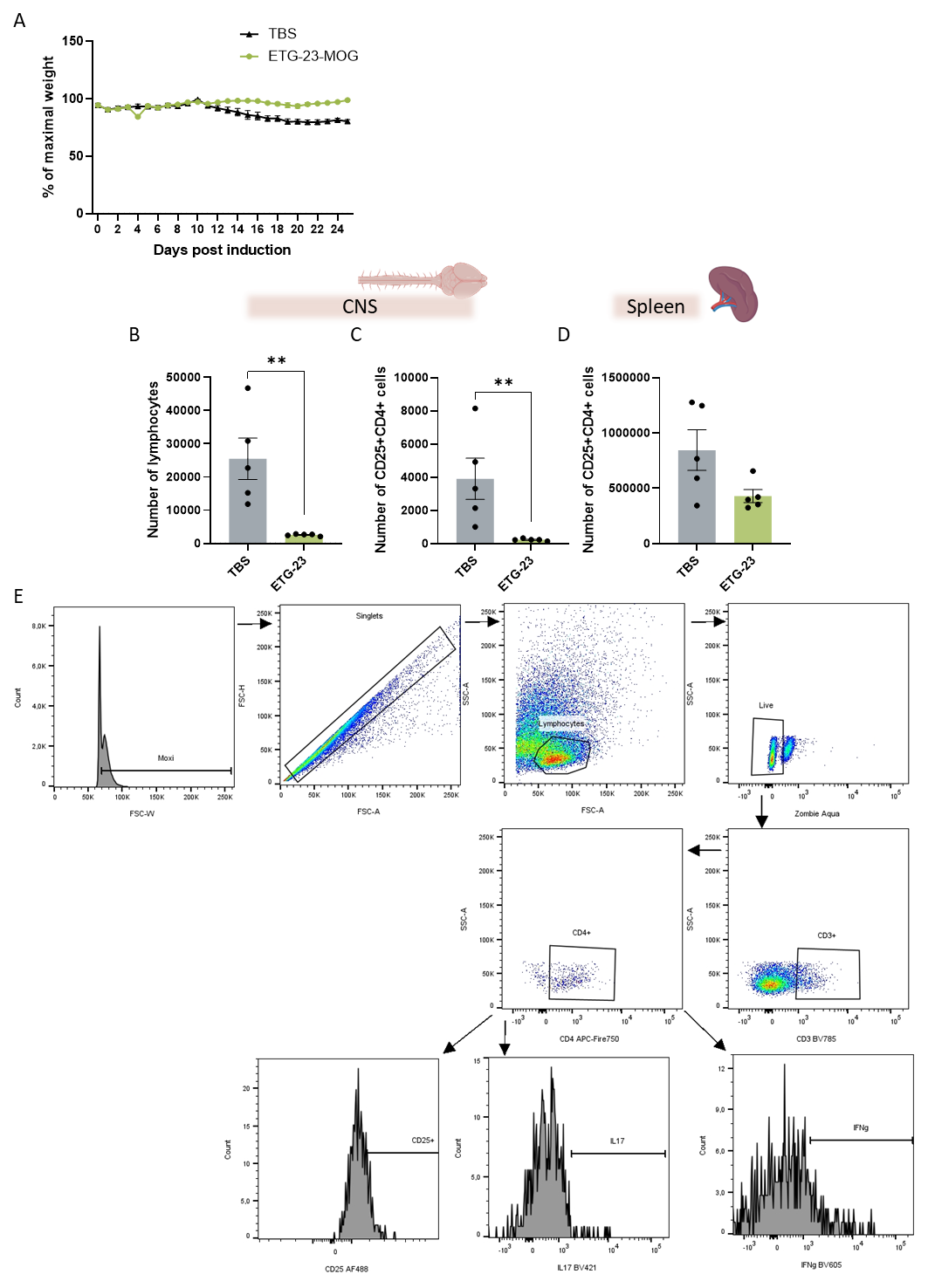


**Figure S3. Weight follow-up and gating of CNS-infiltrating and splenic immune cells. A-E**. On day 13 dpi, immune cells of the CNS and spleen were isolated and counted. After 4 h of stimulation with PMA, CaI and Golgiplug, immune cells were analyzed using flow cytometry for IFN-γ, IL-17 and CD25 expression by CD4^+^ T cells. (**A**) Follow-up weight, as % of maximal weight, of EAE in Figure 1A. (**B-C**) Number of total lymphocytes (**B**) and CD25^+^CD4^+^ T cells (**C**) in the CNS. (**D**) Number of CD25^+^CD4^+^ T cells in the spleen. n=5; Mann-Whitney test compared to TBS. (**E**) Gating strategy. **: p<0.01


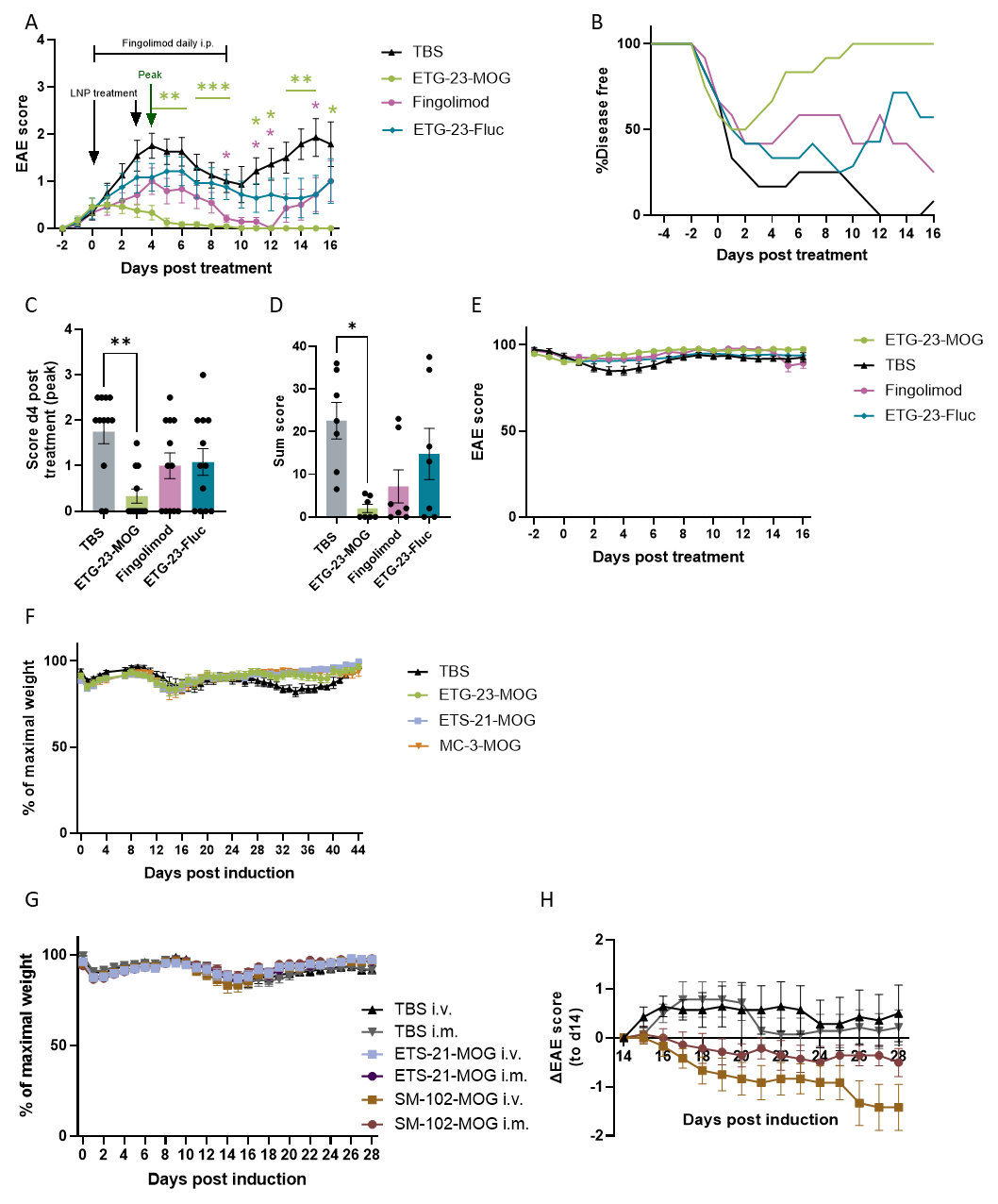


**Figure S4. Follow-up of weight of EAE and T1D. (A-D)** MOG_35-55_ EAE was induced in female C57BL/6 mice. At 7 and 10 dpi, mice were i.v. treated with buffer control or ETG-23 loaded with MOG or Fluc mRNA (5 µg/dose). Mice were weighed (**E**) and scored daily (**A**). Two-way ANOVA with Tukey’s multiple comparison compared to TBS. **(B**) Incidence of disease-free mice. (**C**) Score at peak of disease (19 dpi). (**D**) Sum of scores. n=7; Mann-Whitney test compared to TBS. **(E)** Follow-up weight, as % of maximal weight, of EAE in Figure 1K and Figure S4A-D. (F) Follow-up weight, as % of maximal weight, of EAE in Figure 3A-E. (**G-H**) MOG_35-55_ EAE was induced in female C57BL/6 mice. At 7 and 10 dpi, mice were i.v. or i.m. treated with buffer control or ETS-21 or SM-102 loaded with MOG mRNA (5 µg/dose). (**G**) Follow-up weight, as % of maximal weight, of EAE in Figure 3F-J. (**H**) Daily scoring of EAE mice. ∆ compared to 14 dpi. n=6-7. *: p<0.05; **: p<0.01; ***: p<0.001

**
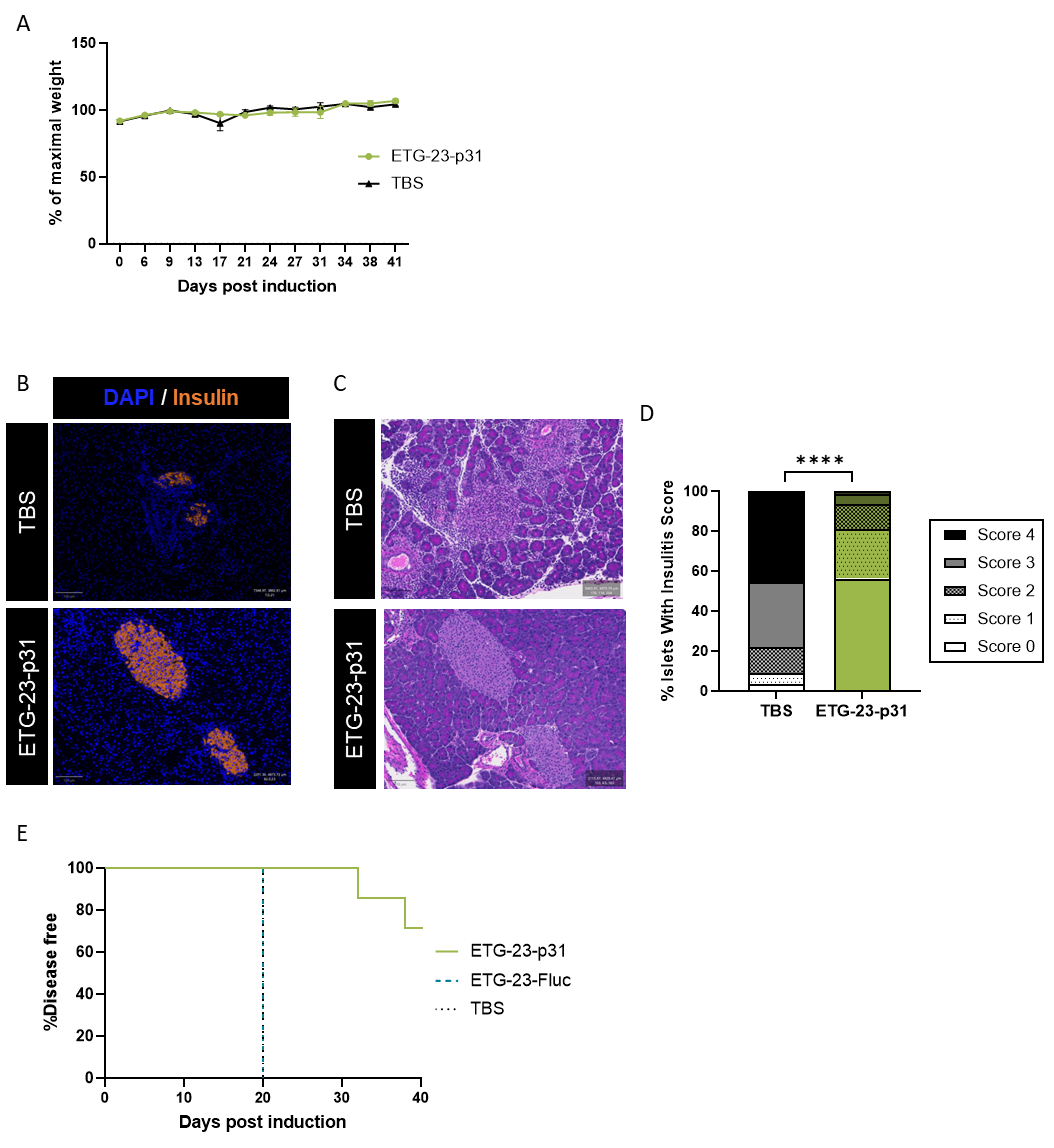
**

**Figure S5. Histology analysis of T1D model showed preserved islet morphology without insulitis after mRNA-LNP treatment.** NOD-SCID mice received CD4^+^ T cells from BDC2.5 transgenic mice to induce diabetes. 1 and 4 days later, recipient mice received i.v. treatment with ETG-23-p31 (5 µg/dose) or buffer control. **(A)** Follow-up weight, as % of maximal weight, of T1D in Figure 2. (**B**) Representative histological examination of insulin revealed that ETG-23-p31 treatment preserved pancreatic islet architecture insulin, whereas the TBS-treated group exhibited marked loss of insulin in the islets. (**C-D**) Insulitis was quantified in H&E-stained pancreatic sections using a 0-4 scoring system (defined in method section). Most islets in TBS displayed severe insulitis (scores 3-4), whereas islets in ETG-23-p31 predominantly showed little to no infiltration (scores 0-1). Scoring was done blindly and a minimum of 15 islets were scored per mouse (5 mice per each group). A Chi-square test was used for statistical analyses of insulitis severity scores. (**E**) Incidence of disease-free mice after treatment with ETG-23-p31, ETG-23-Fluc or TBS. n=8-10

**
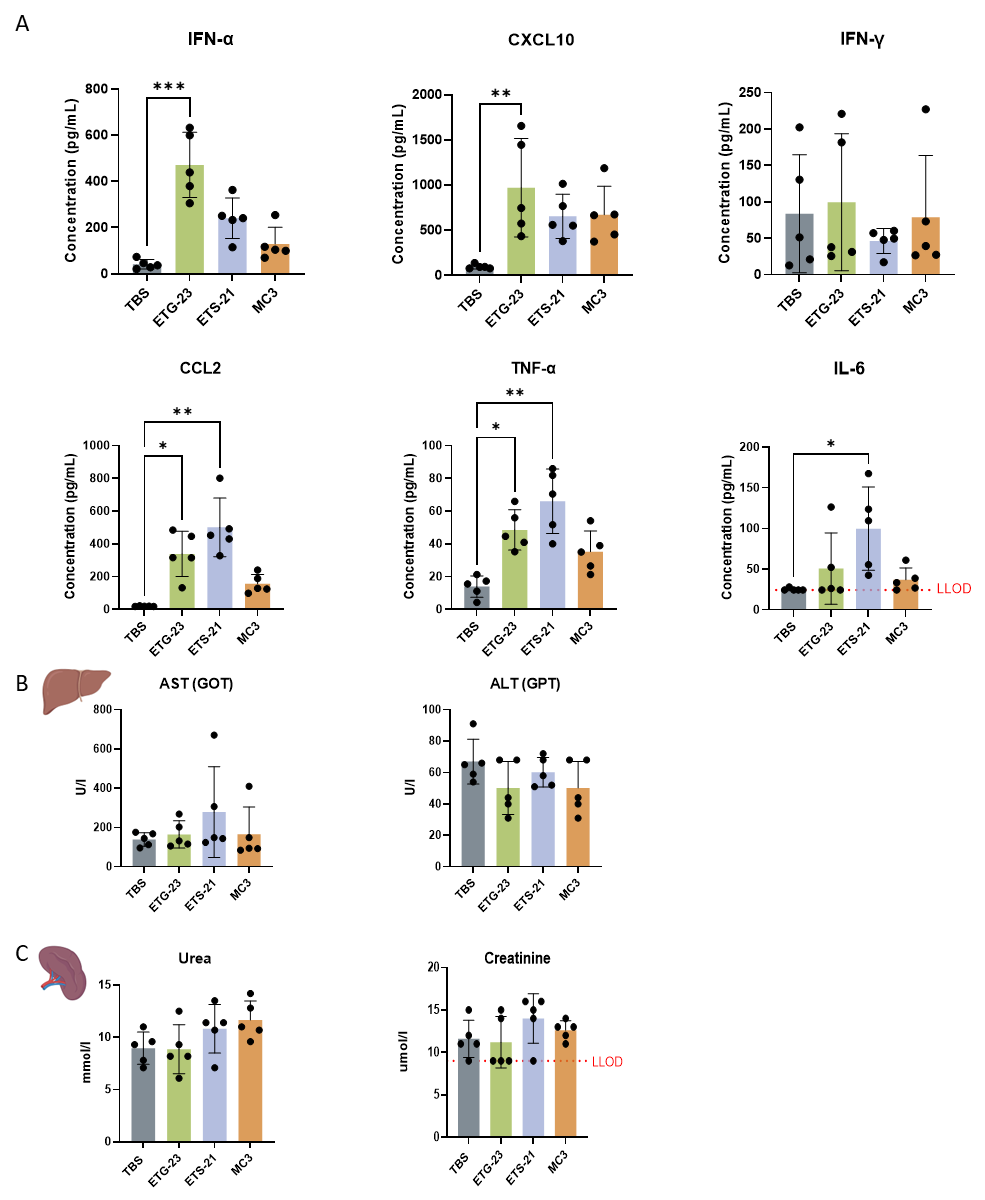
**

**Figure S6. A single high dose LNP treatment is well tolerated in mice.** (**A**) Female Balb/c mice received one i.v. treatment with ETG-23, ETS-21 or MC3 LNP containing CD90.1 mRNA (20 µg/dose) or buffer. Five hours post injection, blood was isolated and cytokines or chemokines were analyzed using LegendPlex. (**B-C**) Female Balb/c mice received one i.v. treatment with ETG-23, ETS-21 or MC3 LNP containing MOG mRNA (60 µg/dose). Liver (**B**) and kidney (**C**) parameters were evaluated in the blood 24 hpi. n=5; Kruskal-Wallis test compared within groups; *: p<0.05; **: p<0.01; ***: p<0.001; LLOD: lower limit of detection


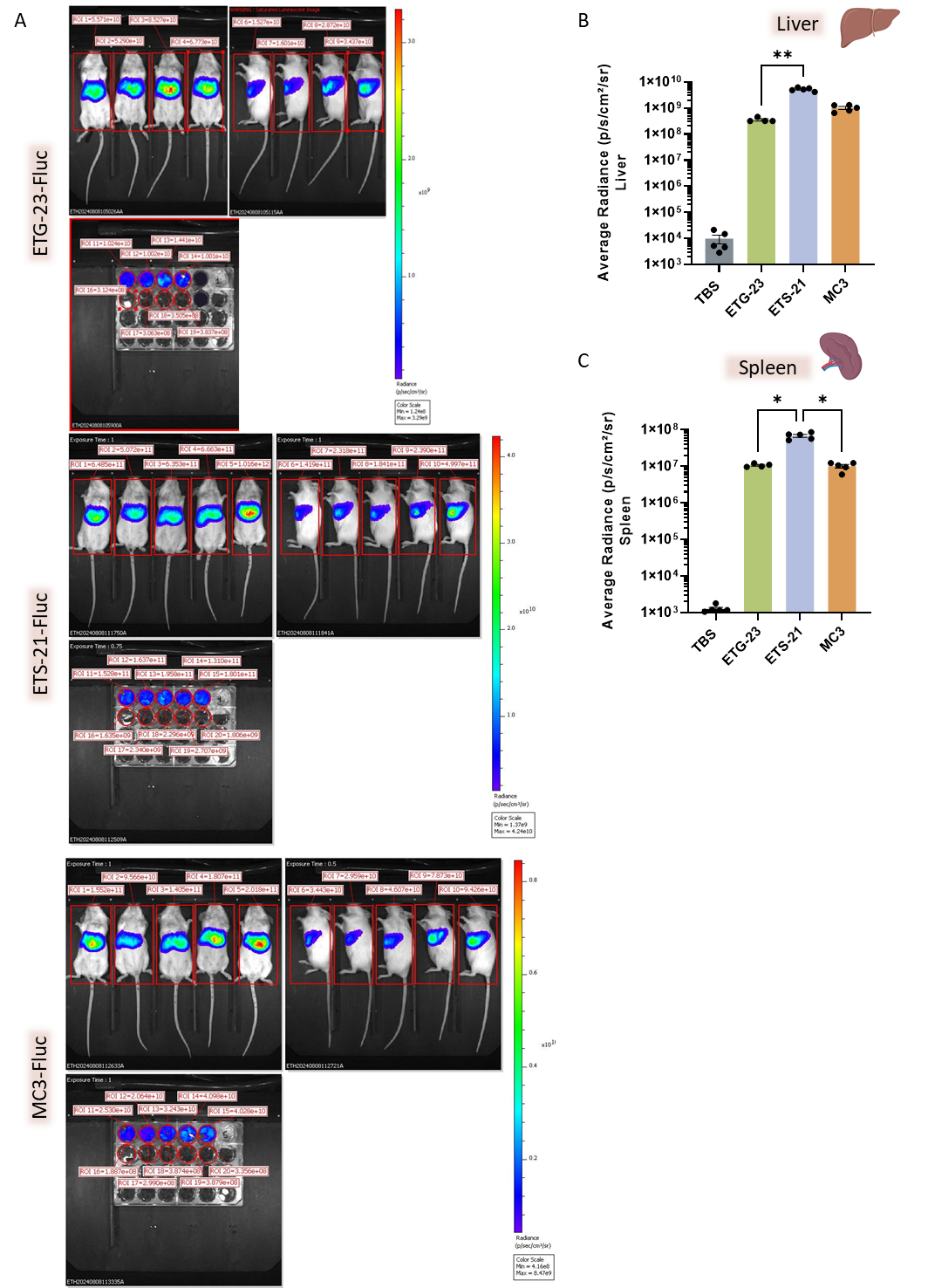


**Figure S7. IVIS images of mice i.v. injected with different LNPs loaded with Fluc mRNA.** Mice were i.v. injected with different LNPs loaded with Fluc mRNA (5 µg/dose). After 24 h, *in vivo* imaging system (IVIS) was performed and Fluc signal was measured. (**A**) Showing representative IVIS images per group. In each plate, the top row contains the liver, while the second row represents the spleen. (**B-C**) Quantification of Fluc signal in liver (**B**) and spleen (**C**). n=4-5. Kruskal-Wallis test within LNP groups. *: p<0.05; **: p<0.01


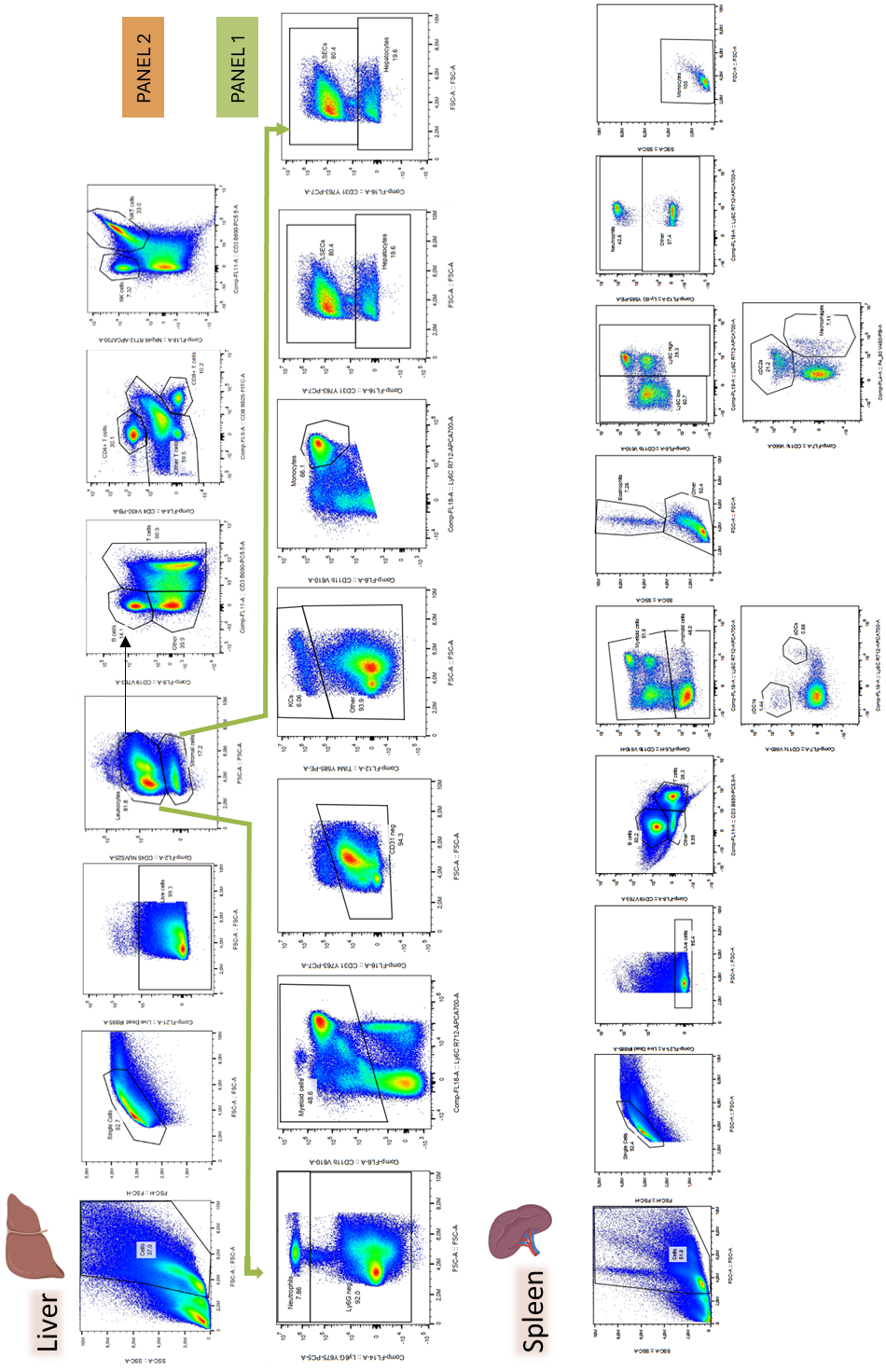


**Figure S8. Gating strategy liver and spleen cells after i.v. LNP administration.** CD90.2 mice were i.v. injected with different LNPs loaded with CD90.1 mRNA (20 µg/dose). After 24 h, CD90.1^+^ cells were isolated from liver and spleen and investigated using flow cytometry. cDC: conventional dendritic cells; KC: Kupffer cells; LSEC: liver sinusoidal endothelial cells; pDC: plasmacytoid dendritic cells.

**
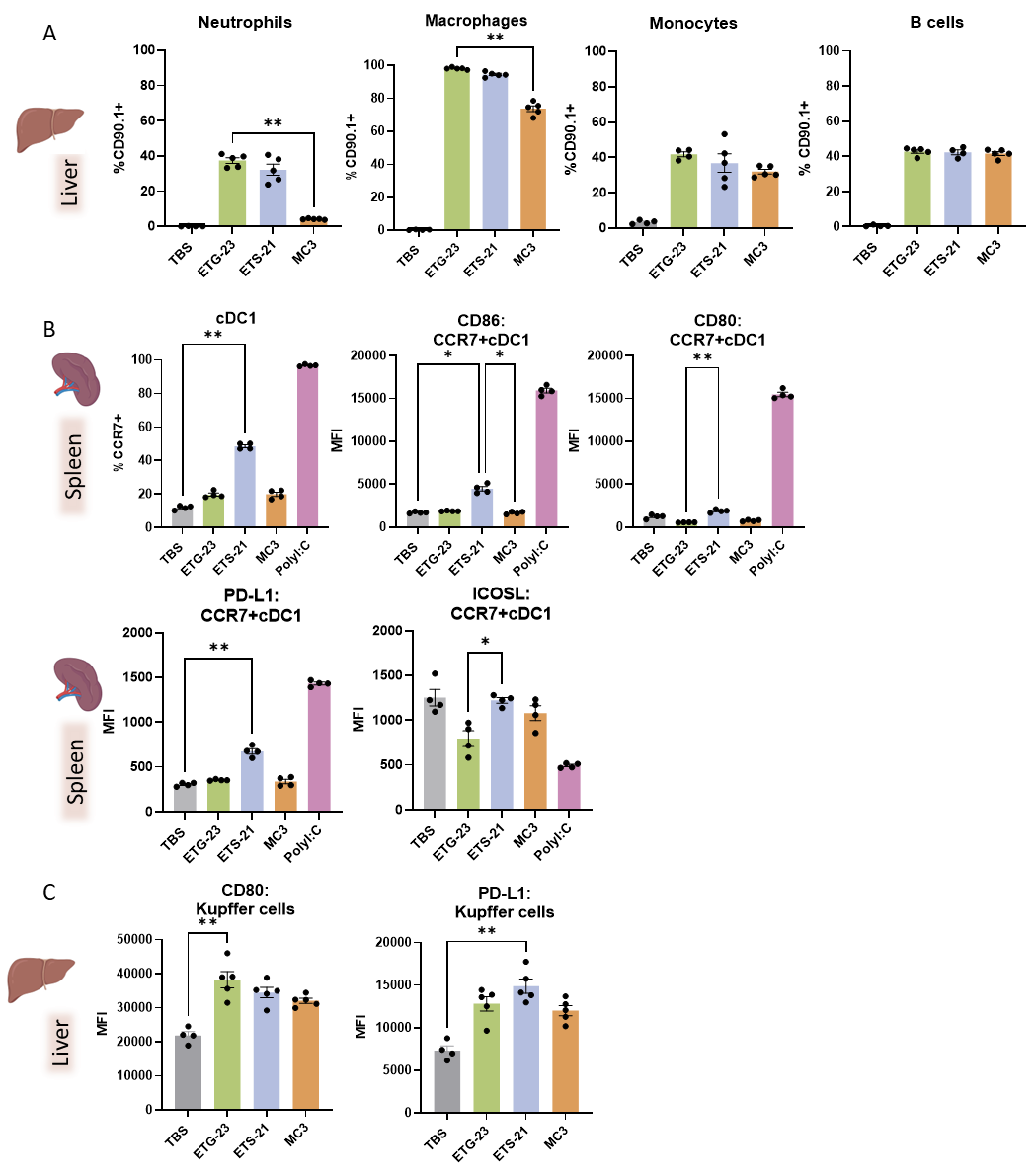
**

**Figure S9**. **LNPs induce a mature, homeostatic phenotype in antigen-presenting cells.** (**A**) CD90.2 mice were i.v. injected with different LNPs loaded with CD90.1 mRNA (20 µg/dose). After 24 h, cells were isolated from the liver and investigated using flow cytometry. Gating strategy in Figure S8. n=3-5, Kruskal-Wallis test compared within LNP groups. (**B**) Mice were i.v. injected with different LNPs loaded with MOG mRNA or PolyI:C (5 µg/dose). After 16h, co-stimulatory markers CD86 and CD80, immunogenic marker PD-L1 and homeostatic molecule ICOSL of cDC isolated from the spleen were investigated using flow cytometry. Kruskal-Wallis test compared within groups excluding PolyI:C. Gating strategy in Figure S13. (**C**) Mice were i.v. injected with CD90.1 mRNA (20 µg/dose). After 24 h, co-stimulatory marker CD80 and immunogenic marker PD-L1 were investigated in Kupffer cells of the liver using flow cytometry. Gating strategy in Figure S8. Kruskal-Wallis test within groups. *: p<0.05; **: p<0.01; cDC: conventional dendritic cells; PolyI:C: polyinosinic-polycytidylic acid


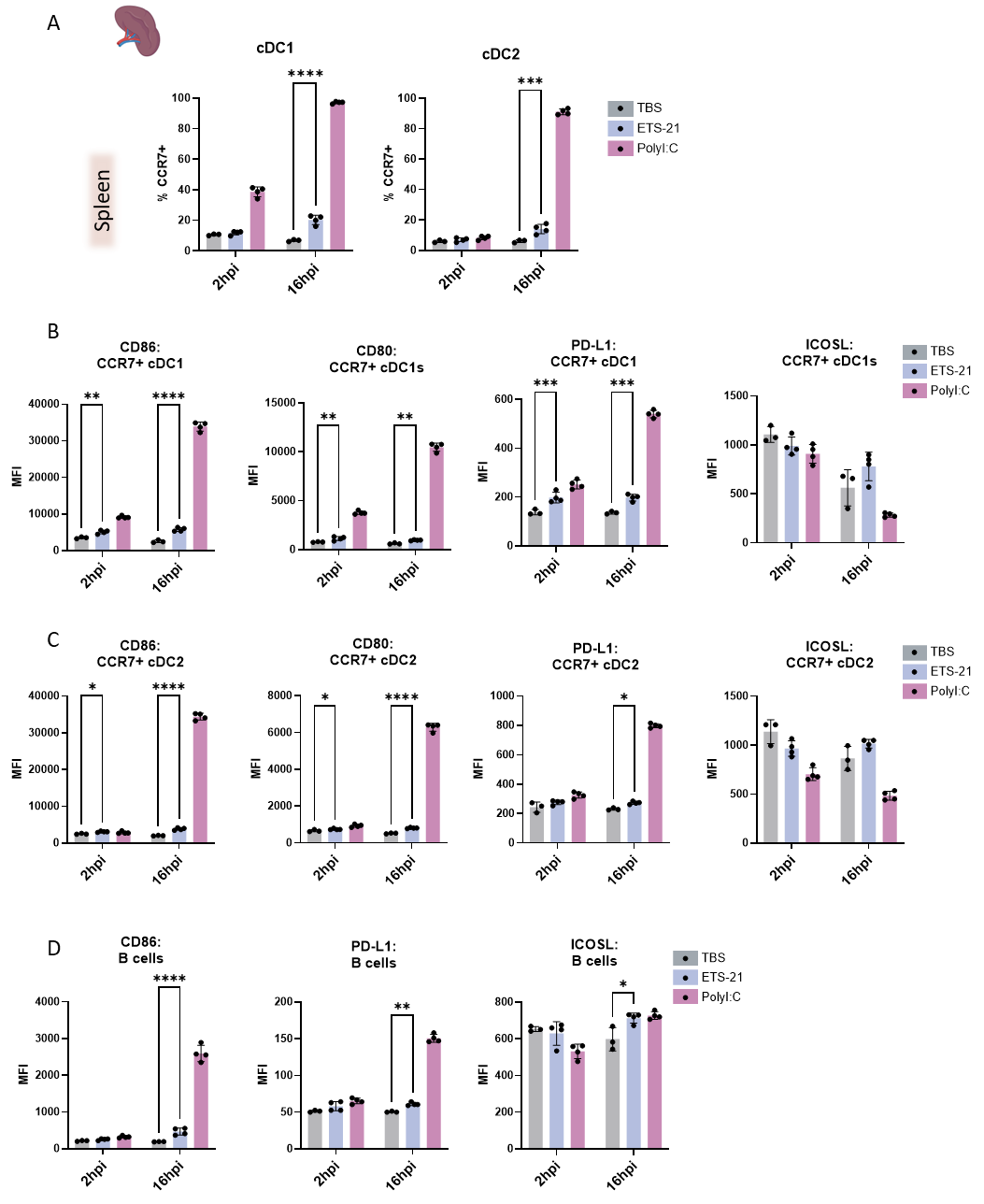


**Figure S10. mRNA-LNPs induce a mature, homeostatic phenotype in APCs.** (**A-D**) Mice were i.v. injected with different LNPs loaded with MOG mRNA or PolyI:C in ETS-21 (5 µg/dose). After 2 h or 16h, co-stimulatory markers CD86 and CD80, immunogenic marker PD-L1 and homeostatic molecule ICOSL in CCR7^+^ cDC (**A-C**) and B cells (**D**) were investigated in the spleen using flow cytometry. Gating strategy in Figure S13. Two-way ANOVA with Šidák multiple comparison test was used to compare groups within each timepoint excluding PolyI:C. n=3-4; *: p<0.05; **: p<0.01; ***: p<0.001; ****: p<0.0001; cDC: conventional dendritic cells; PolyI:C: polyinosinic-polycytidylic acid


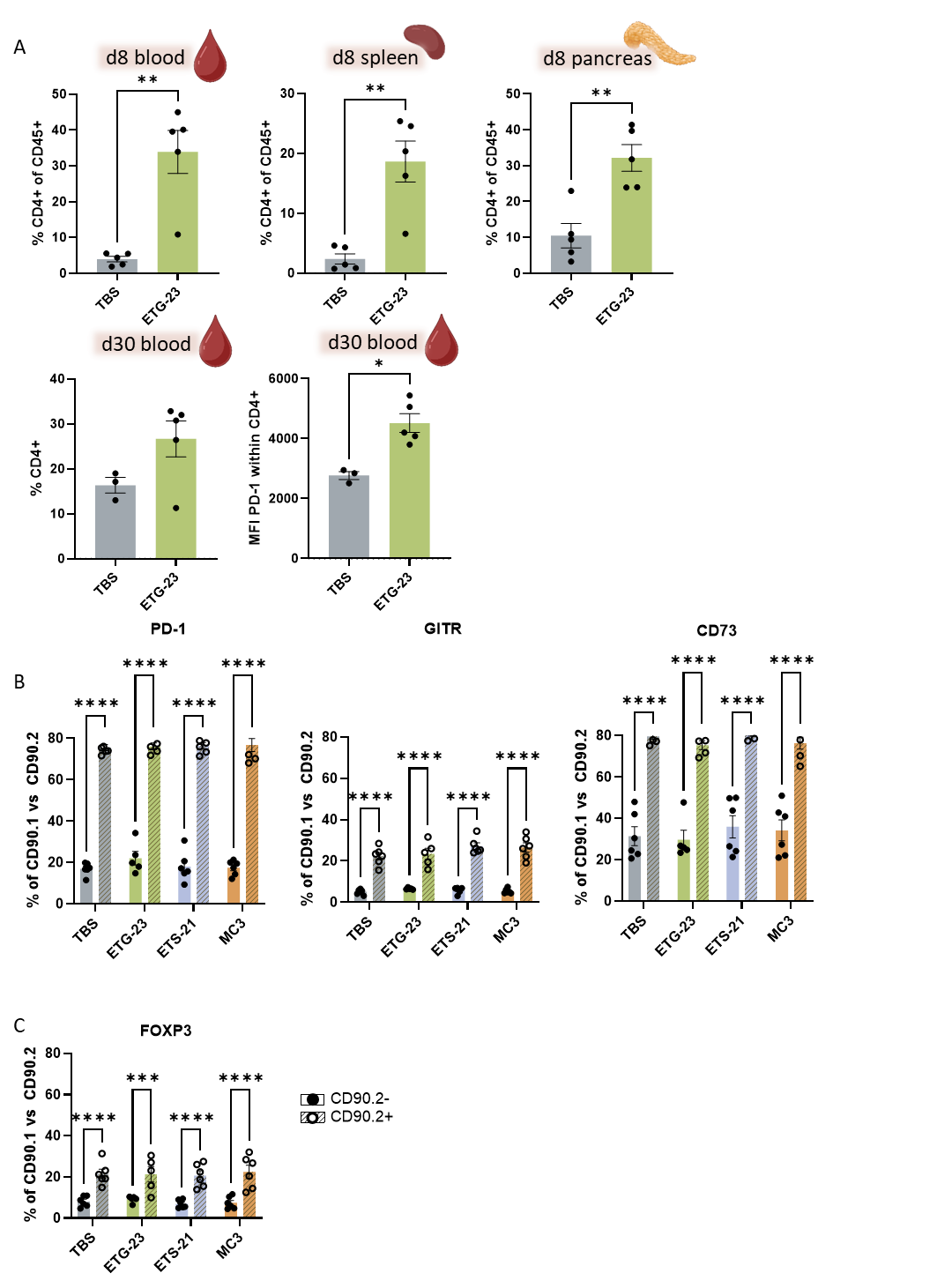


**Figure S11. Induction of exhaustion and anti-inflammatory markers in antigen-specific CD4^+^ T cells by autoantigen mRNA-LNPs.** (**A**) NOD-SCID mice received CD4^+^ T cells from BDC2.5 transgenic mice to induce diabetes. After 1 and 4 days, recipient mice received i.v. treatment with ETG-23-p31 (5 µg/dose) or buffer control. On 8 or 30 dpi, cells of the blood, spleen or pancreas were isolated. Immune cells were analyzed using flow cytometry for CD4 or PD-1 (exhaustion) expression. Gating strategy in Figure S15. n=3-5; Mann-Whitney test. (**B-C**) CD4^+^ T cells were isolated from 2D2 transgenic mice (CD90.2 background), and i.v. injected in recipient mice (CD90.1 background). The next day, MOG_35-55_ EAE was induced in recipient mice. On day 7 and 10, mice were i.v. injected with buffer control or different LNPs loaded with MOG mRNA (5 µg/dose). After 3 days, splenocytes were isolated and studied using flow cytometry after 4 h stimulation with PMA, CaI and Golgiplug. Analysis of exhaustion markers PD-1, GITR, CD73 (**B**) and anti-inflammatory marker FOXP3 (**C**) within both CD90.2 positive and negative cells. Gating strategy in Figure S14. n=5-6; Two-way ANOVA test; *: p<0.05; **: p<0.01; ***: p<0.001; ****: p<0.0001

**
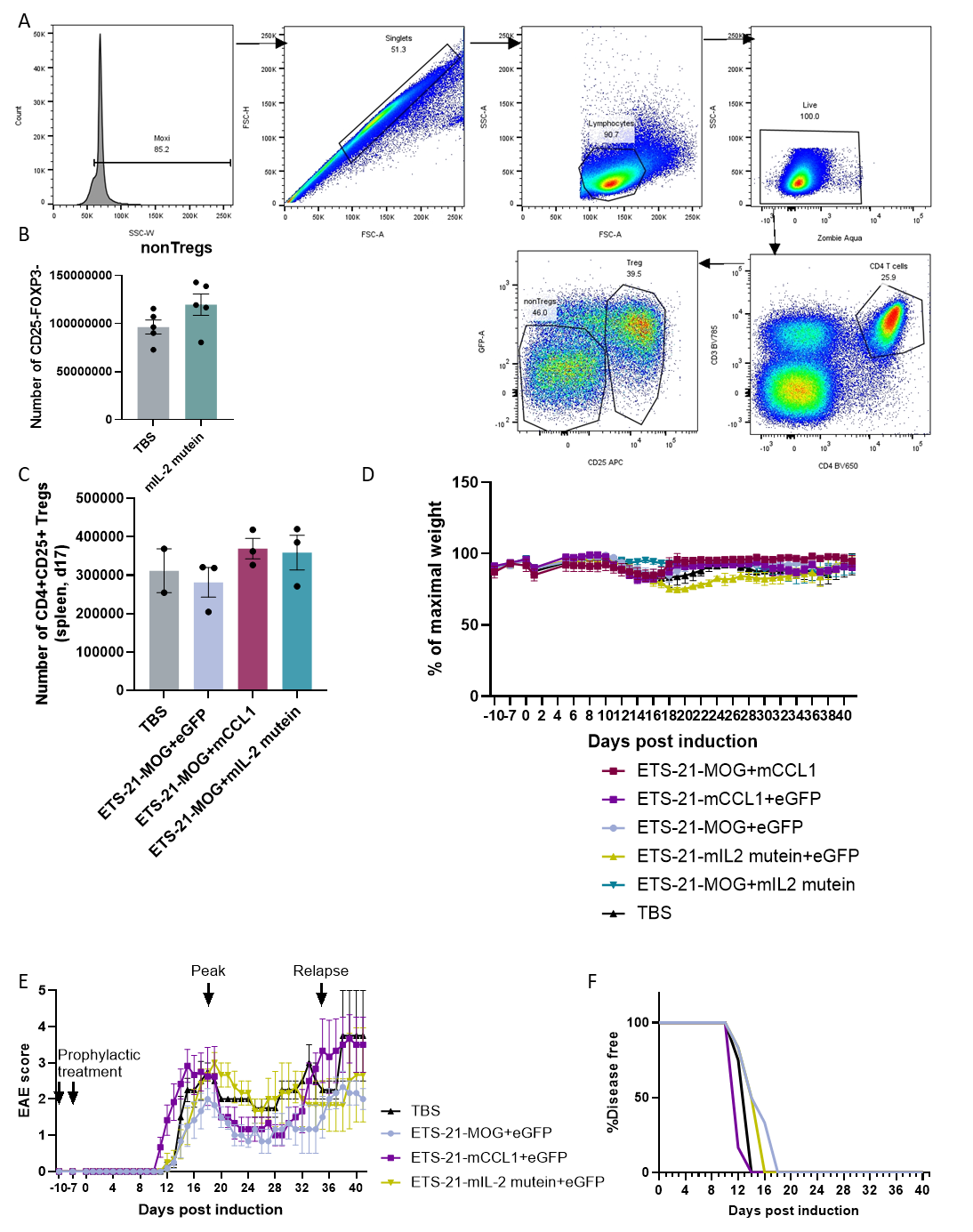
**

**Figure S12. Prophylactic treatment with co-delivered autoantigen and immunoregulatory mRNA dampen disease severity.** (**A**) Gating strategy of Tregs after i.v. injection into FOXP3 reporter mice. (**B**) Immune cells from the spleen were isolated and nonTreg numbers were studied using flow cytometry. Kruskal-Wallis test; n=5. (**C-F**) Female C57BL/6 mice were i.v. injected with ETS-21 loaded with MOG and/or immunoregulatory mRNA (5 µg/dose) or buffer control at 10 and 7 days before active MOG_35-55_ EAE induction. (**C**) At peak (d17), immune cells of the spleen were isolated and counted. Splenocytes were analyzed using flow cytometry for CD25 expression by CD4 cells. Gating strategy in Figure S3. n=2-3. (**D**) Follow-up weight, as % of maximal weight, of EAE in Figure 6. (**E**) Mice were scored daily. (**F**) Incidence of disease-free mice. Data from this Figure and Figure 6 were from the same experiment; Kruskal-Wallis test within shown groups. n=3-6


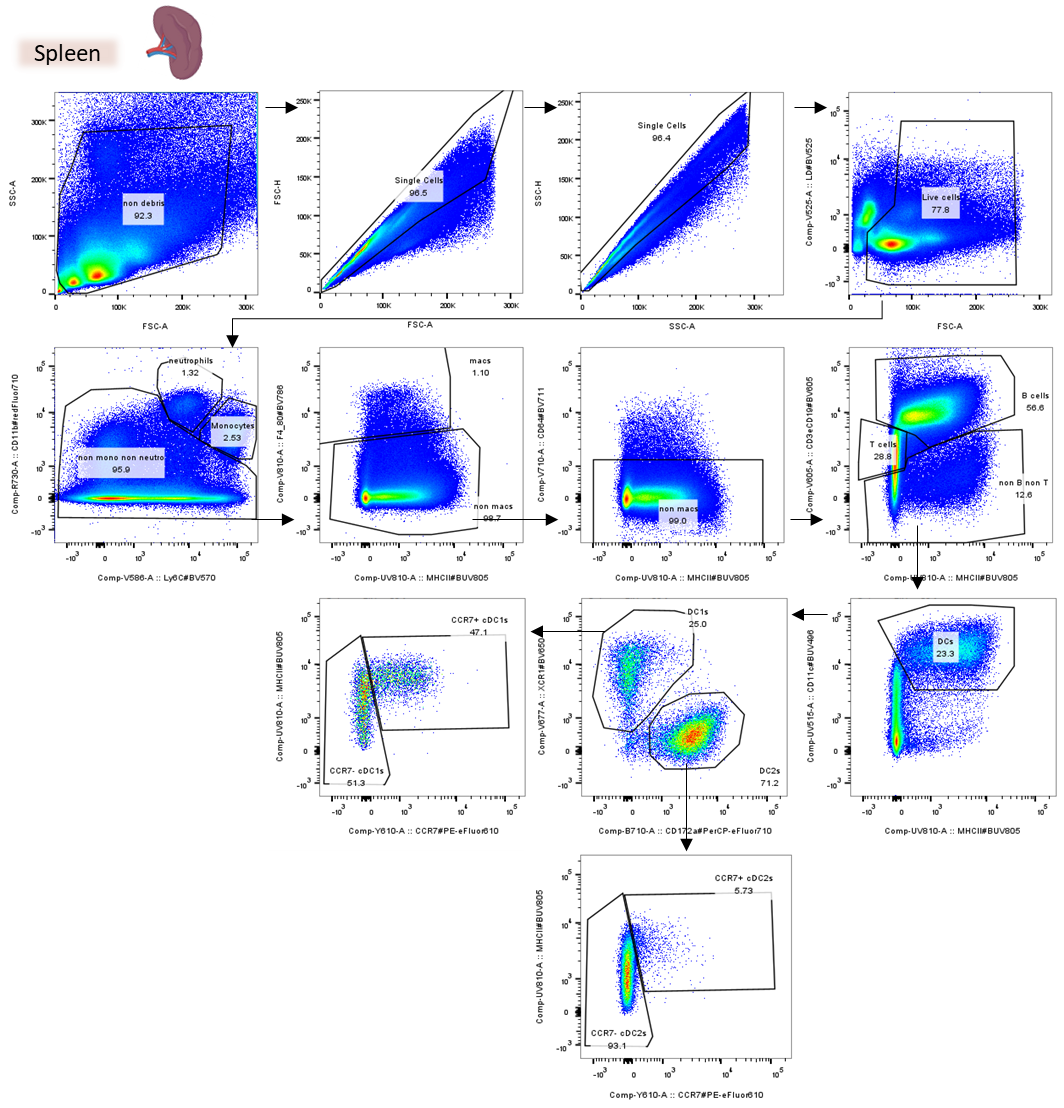


**Figure S13**. **Gating strategy of APC in spleen.**


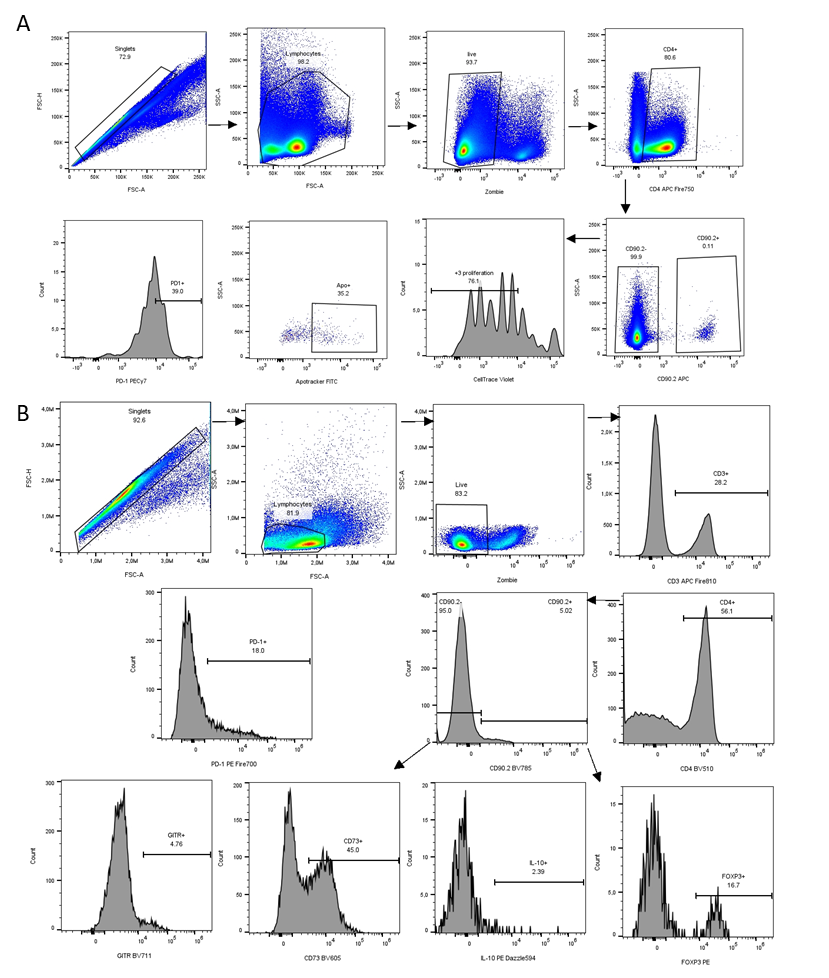


**Figure S14. Gating strategy of CD90.2 positive and negative cells**. (**A**) CD4^+^ T cells were isolated from 2D2 transgenic mice (CD90.2 background), labelled with CellTrace Violet and i.v. injected in recipient mice (CD90.1 background). The next day, mice were i.v. injected with buffer control or with different LNPs loaded with MOG mRNA (5 µg/dose). After 4 days, splenocytes and CD4^+^ T cells were isolated and studied using flow cytometry. Representative gates of CD90.2^+^ vs CD90.2^-^ cells, %proliferation of more than 3 divisions were evaluated, %apoptotic^+^, %PD-1^+^ of CD90.2^+^ cells. (**B**) CD90.2^+^ CD4^+^ T cells were isolated from 2D2 transgenic mice, and i.v. injected in CD90.2^-^ recipient mice. The next day, MOG_35-55_ EAE was induced in the recipient mice. On day 7 and 10, mice were i.v. injected with buffer control or different LNPs loaded with MOG mRNA (5 µg/dose). After 3 days, splenocytes were isolated and studied using flow cytometry after 4 h stimulation with PMA, CaI and Golgiplug. Representative plots of CD90.2^+^ vs CD90.2^-^ cells, %PD-1^+^, %GITR^+^, %CD73^+^, %IL-10^+^ and %FOXP3^+^ CD90.2^+^ cells.

**
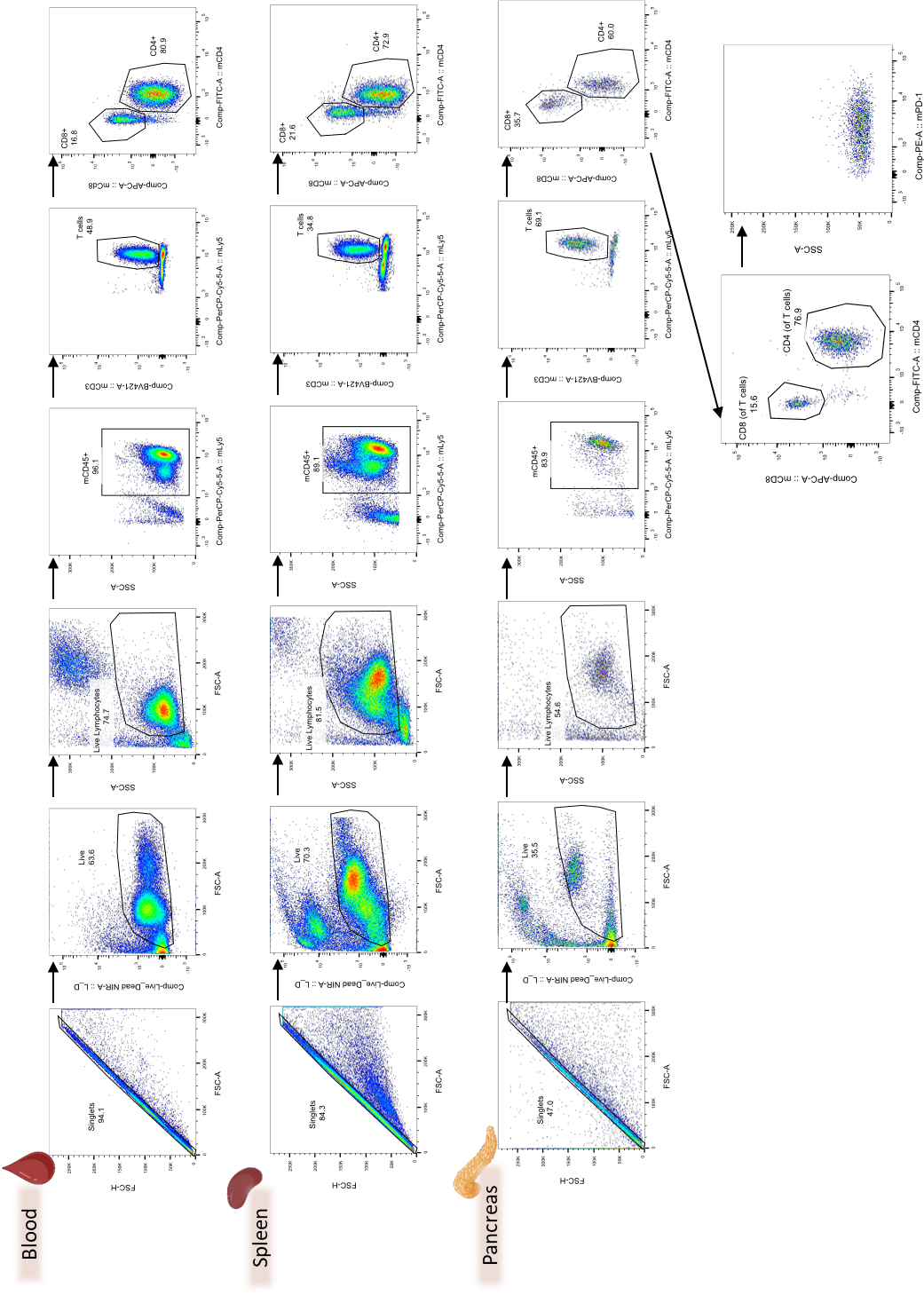
**

**Figure S15. Gating strategy of immune cells in the blood, spleen and pancreas.**

**
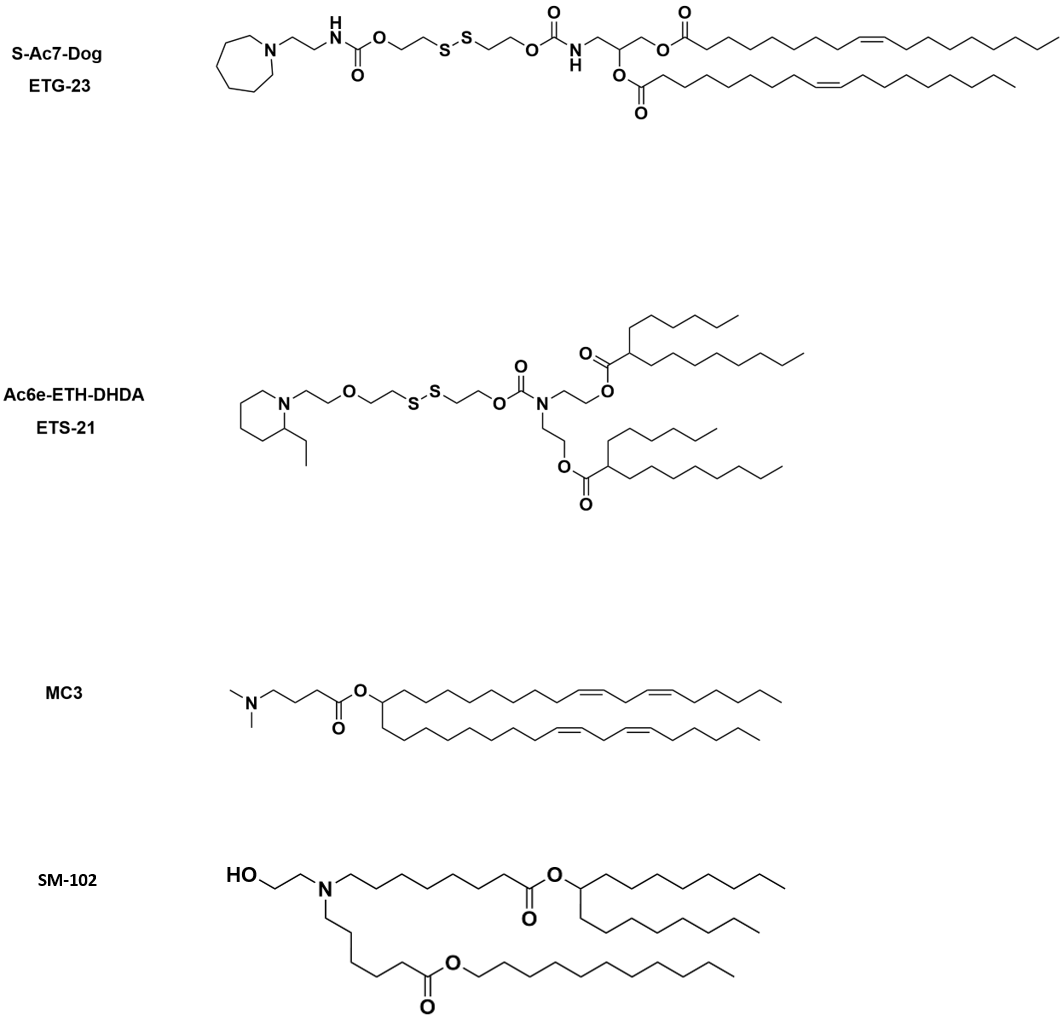
**

**Figure S16: Chemical structure of ionizable LNPs.**

**Table S1. mRNA sequences used**

| **mRNA Name** | **mRNA Sequence** |
| --- | --- |
| mCCL1-MSA | AGGCCGGCGGGUUUCUGACAUCCGGCGGGUUUCUGACAUCCGGCGGGUUUCUGACAUCCGGCGGGUUUCUGACAUCCGGCGGGUUUCUGACAUCCGGCGGGUUUCUGACAUCCGGCGGGUUUCUGACAUCCGGCGGGUUUCUGACAUCCGGCGGGUUUCUGACAUCCGGCGGGUUUCUGACAUUCACAACCAGGCCUCCACAACCAUGAAGUGGGUGACCUUCCUGCUGCUGCUGUUCGUGAGCGGCAGCGCCUUCAGCAGGGGCGUGUUCAGGAGGGAGGCCCACAAGAGCGAGAUCGCCCACAGGUACAACGACCUGGGCGAGCAGCACUUCAAGGGCCUGGUGCUGAUCGCCUUCAGCCAGUAUCUCCAGAAGUGCAGCUACGACGAGCACGCCAAGCUGGUGCAGGAGGUGACCGACUUCGCCAAGACAUGUGUGGCCGACGAGAGCGCCGCCAACUGCGACAAGAGCCUGCACACCCUGUUCGGCGACAAGCUGUGCGCCAUCCCCAACCUGAGGGAGAACUACGGCGAGCUGGCCGACUGCUGCACCAAGCAGGAGCCCGAGAGGAACGAGUGCUUCCUGCAGCACAAGGACGACAACCCCAGCCUGCCCCCCUUCGAGAGGCCCGAGGCCGAGGCCAUGUGCACCAGCUUCAAGGAGAACCCCACCACCUUCAUGGGCCACUAUCUCCACGAGGUGGCCAGGAGGCACCCCUACUUCUACGCCCCCGAGCUGCUGUACUACGCCGAGCAGUACAACGAGAUCCUGACCCAGUGCUGCGCCGAGGCCGACAAGGAGAGCUGCCUGACCCCCAAGCUGGACGGCGUGAAGGAGAAGGCCCUGGUGAGCAGCGUGAGGCAGAGGAUGAAGUGCAGCAGCAUGCAGAAGUUCGGCGAGAGGGCCUUCAAGGCCUGGGCCGUGGCCAGGCUGAGCCAGACCUUCCCCAACGCCGACUUCGCCGAGAUCACCAAGCUGGCCACCGACCUGACCAAGGUGAACAAGGAGUGCUGCCACGGCGAUCUCCUGGAGUGCGCCGACGACAGGGCCGAGCUGGCCAAGUACAUGUGCGAGAACCAGGCCACCAUCAGCAGCAAGCUGCAGACAUGUUGCGACAAGCCCCUGCUGAAGAAGGCCCACUGCCUGAGCGAGGUGGAGCACGACACCAUGCCCGCCGAUCUCCCCGCCAUCGCCGCCGACUUCGUGGAGGACCAGGAGGUGUGCAAGAACUACGCCGAGGCCAAGGACGUGUUCCUGGGCACCUUCCUGUACGAGUACAGCAGGAGGCACCCCGACUACAGCGUGAGCCUGCUGCUGAGGCUGGCCAAGAAGUACGAGGCCACCCUGGAGAAGUGCUGCGCCGAGGCCAACCCCCCCGCCUGCUACGGCACCGUGCUGGCCGAGUUCCAGCCCCUGGUGGAGGAGCCCAAGAACCUGGUGAAGACCAACUGCGACCUGUACGAGAAGCUGGGCGAGUACGGCUUCCAGAACGCCAUCCUGGUGAGGUACACCCAGAAGGCCCCCCAGGUGAGCACCCCCACCCUGGUGGAGGCCGCCAGGAACCUGGGCAGGGUGGGCACCAAGUGCUGCACCCUGCCCGAGGACCAGAGGCUGCCCUGCGUGGAGGACUACCUGAGCGCCAUCCUGAACAGGGUGUGCCUGCUGCACGAGAAGACCCCCGUGAGCGAGCACGUGACCAAGUGCUGCAGCGGCAGCCUGGUGGAGAGGAGGCCCUGCUUCAGCGCCCUGACCGUGGACGAGACCUACGUGCCCAAGGAGUUCAAGGCCGAGACCUUCACCUUCCACAGCGACAUCUGCACCCUGCCCGAGAAGGAGAAGCAGAUCAAGAAGCAGACCGCCCUGGCCGAGCUGGUGAAGCACAAGCCCAAGGCCACCGCCGAGCAGCUGAAGACCGUGAUGGACGACUUCGCCCAGUUCCUGGACACAUGUUGCAAGGCCGCCGACAAGGACACAUGUUUCAGCACCGAGGGCCCCAACCUGGUGACCAGGUGCAAGGACGCCCUGGCCGGCGGCGGCGGCAGCGGCGGCGGCGGCAGCGGCGGCGGCGGCAGCAAGAGCAUGCUGACCGUGAGCAACAGCUGCUGCCUGAACACCCUGAAGAAGGAGCUGCCCCUGAAGUUCAUCCAGUGCUACAGGAAGAUGGGCAGCAGCUGCCCCGACCCCCCCGCCGUGGUGUUCAGGCUGAACAAGGGCAGGGAGAGCUGCGCCAGCACCAACAAGACCUGGGUGCAGAACCACCUGAAGAAGGUGAACCCCUGCUAAACUCGAGUGUUUUGGCUGGGUUUUUCCUUGUUCGCACCGGACACCUCCAGUGACCAGACGGCAAGGUUUUUAUCCCAGUGUAUAUUGUCGACAAAAAAAAAAAAAAAAAAAAAAAAAAAAAAAAAAAAAAAAAAAAAAAAAAAAAAAAAAAAAAAAAAAAAAAAAAAAAAAAAAAAAAAAAAAAAA |
| CD90.1 | AGGAGUGUGAAAUCUUCAGAGAAGAAUUUCUCUUUAGUUCUUUGCAAGAAGGUAGAGAUAAAGACACUUUUUCAAAAGCCACCAUGAACCCCGCCAUCAGCGUGGCCCUGCUGCUGAGCGUGCUCCAAGUGAGCAGGGGCCAGAAGGUGACCAGCCUGACCGCCUGCCUGGUGAACCAGAACCUGAGGCUGGACUGCAGGCACGAGAACAACACCAAGGACAACAGCAUCCAGCACGAGUUCAGCCUGACCAGGGAGAAGAGGAAGCACGUGCUGAGCGGCACCCUGGGCAUCCCCGAGCACACCUACAGGAGCAGGGUGACCCUGAGCAACCAGCCCUACAUCAAGGUGCUGACCCUGGCCAACUUCACCACCAAGGACGAGGGCGACUACUUCUGCGAGCUGAGGGUGAGCGGCGCCAACCCCAUGAGCAGCAACAAGAGCAUCAGCGUGUACAGGGACAAGCUGGUGAAGUGCGGCGGCAUCAGCCUGCUGGUGCAGAACACCAGCUGGAUGCUGCUGCUGCUGCUGAGCCUGAGCCUGCUGCAGGCCCUGGACUUCAUCAGCCUGUAAACUCGAGGCUGGAGCCUCGGUGGCCUAGCUUCUUGCCCCUUGGGCCUCCCCCCAGCCCCUCCUCCCCUUCCUGCACCCGUACCCCCGUGGUCUUUGAAUAAAGUCUGAGUGGGCGGCAGUCGACAAAAAAAAAAAAAAAAAAAAAAAAAAAAAAAAAAAAAAAAAAAAAAAAAAAAAAAAAAAAAAAAAAAAAAAAAAAAAAAAAAAAAAAAAA |
| eGFP | AGGCCGGCGGGUUUCUGACAUCCGGCGGGUUUCUGACAUCCGGCGGGUUUCUGACAUCCGGCGGGUUUCUGACAUCCGGCGGGUUUCUGACAUCCGGCGGGUUUCUGACAUCCGGCGGGUUUCUGACAUCCGGCGGGUUUCUGACAUCCGGCGGGUUUCUGACAUCCGGCGGGUUUCUGACAUUCACAACCAGGCCUCCACAACCAUGGUGAGCAAGGGCGAGGAGCUGUUCACCGGGGUGGUGCCCAUCCUGGUCGAGCUGGACGGCGACGUAAACGGCCACAAGUUCAGCGUGUCCGGCGAGGGCGAGGGCGAUGCCACCUACGGCAAGCUGACCCUGAAGUUCAUCUGCACCACCGGCAAGCUGCCCGUGCCCUGGCCCACCCUCGUGACCACCCUGACCUACGGCGUGCAGUCUCGAGUGUUUUGGCUGGGUUUUUCCUUGUUCGCACCGGACACCUCCAGUGACCAGACGGCAAGGUUUUUAUCCCAGUGUAUAUUGUCGACAAAAAAAAAAAAAAAAAAAAAAAAAAAAAAAAAAAAAAAAAAAAAAAAAAAAAAAAAAAAAAAAAAAAAAAAAAAAAAAAAAAAAAAAAAAAAA |
| Fluc | AGGCCGGCGGGUUUCUGACAUCCGGCGGGUUUCUGACAUCCGGCGGGUUUCUGACAUCCGGCGGGUUUCUGACAUCCGGCGGGUUUCUGACAUCCGGCGGGUUUCUGACAUCCGGCGGGUUUCUGACAUCCGGCGGGUUUCUGACAUCCGGCGGGUUUCUGACAUCCGGCGGGUUUCUGACAUUCACAACCAGGCCUCCACAACCAUGGAGGACGCCAAGAACAUCAAGAAGGGCCCCGCCCCCUUCUACCCCCUGGAGGACGGCACCGCCGGCGAGCAGCUGCACAAGGCCAUGAAGCGGUACGCCCUGGUGCCCGGCACCAUCGCCUUCACCGACGCCCACAUCGAGGUGAACAUCACCUACGCCGAGUACUUCGAGAUGAGCGUGCGGCUGGCCGAGGCCAUGAAGCGGUACGGCCUGAACACCAACCACCGGAUCGUGGUGUGCAGCGAGAACAGCCUGCAGUUCUUCAUGCCCGUGCUGGGCGCCCUGUUCAUCGGCGUGGCCGUGGCCCCCGCCAACGACAUCUACAACGAGCGGGAGCUGCUGAACAGCAUGAACAUCAGCCAGCCCACCGUGGUGUUCGUGAGCAAGAAGGGCCUGCAGAAGAUCCUGAACGUGCAGAAGAAGCUGCCCAUCAUCCAGAAGAUCAUCAUCAUGGACAGCAAGACCGACUACCAGGGCUUCCAGAGCAUGUACACCUUCGUGACCAGCCAUCUCCCCCCCGGCUUCAACGAGUACGACUUCGUGCCCGAGAGCUUCGACCGGGACAAGACCAUCGCCCUGAUCAUGAACAGCAGCGGCAGCACCGGCCUGCCCAAGGGCGUGGCCCUGCCCCACCGGGCCCUGUGCGUGCGGUUCAGCCACGCCCGGGACCCCAUCUUCGGCAACCAGAUCGCCCCCGACACCGCCAUCCUGAGCGUGGUGCCCUUCCACCACGGCUUCGGCAUGUUCACCACCCUGGGCUACCUGAUCUGCGGCUUCCGGGUGGUGCUGAUGUACCGGUUCGAGGAGGAGCUGUUCCUGCGGAGCCUGCAGGACUACAAGAUCCAGACCGCCCUGCUGGUGCCCACCCUGUUCAGCUUCCUGGCCAAGAGCACCCUGAUCGACAAGUACGACCUGAGCAAUCUCCACGAGAUCGCCAGCGGCGGCGCCCCCCUGAGCAAGGAGGUGGGCGAGGCCGUGGCCAAGCGGUUCCAUCUCCCCGGCAUCCGGCAGGGCUACGGCCUGACCGAGACCACCAGCGCCAUCCUGAUCACCCCCAAGGGCGACGACAAGCCCGGCGCCGUGGGCAAGGUGGUGCCCUUCUUCGAGGCCAAGGUGGUGGACCUGGACACCGGCAAGACCCUGGGCGUGAACCAGCGGGGCGAGCUGUGCGUGCGGGGCCCCAUGAUCAUGAGCGGCUACGUGAACAACCCCGAGGCCACCAACGCCCUGAUCGACAAGGACGGCUGGCUGCACAGCGGCGACAUCGCCUACUGGGACGAGGACGAGCACUUCUUCAUCGUGGACCGGCUGAAGAGCCUGAUCAAGUACAAGGGCUACCAGGUGGCCCCCGCCGAGCUGGAGAGCAUCCUGCUGCAGCACCCCAACAUCUUCGACGCCGGCGUGGCCGGCCUGCCCGACGACGACGCCGGCGAGCUGCCCGCCGCCGUGGUGGUGCUGGAGCACGGCAAGACCAUGACCGAGAAGGAGAUCGUGGACUACGUGGCCAGCCAGGUGACCACCGCCAAGAAGCUGCGGGGCGGCGUGGUGUUCGUGGACGAGGUGCCCAAGGGCCUGACCGGCAAGCUGGACGCCCGGAAGAUCCGGGAGAUCCUGAUCAAGGCCAAGAAGGGCGGCAAGAGCAAGCUGUAAACUCGAGUGUUUUGGCUGGGUUUUUCCUUGUUCGCACCGGACACCUCCAGUGACCAGACGGCAAGGUUUUUAUCCCAGUGUAUAUUGUCGACAAAAAAAAAAAAAAAAAAAAAAAAAAAAAAAAAAAAAAAAAAAAAAAAAAAAAAAAAAAAAAAAAAAAAAAAAAAAAAAAAAAAAAAAAA |
| mIL2 mutein | AGGCCGGCGGGUUUCUGACAUCCGGCGGGUUUCUGACAUCCGGCGGGUUUCUGACAUCCGGCGGGUUUCUGACAUCCGGCGGGUUUCUGACAUCCGGCGGGUUUCUGACAUCCGGCGGGUUUCUGACAUCCGGCGGGUUUCUGACAUCCGGCGGGUUUCUGACAUCCGGCGGGUUUCUGACAUUCACAACCAGGCCUCCACAACCAUGAAGUGGGUGACCUUCCUGCUGCUGCUGUUCGUGAGCGGCAGCGCCUUCAGCAGGGGCGUGUUCAGGAGGGAGGCCCACAAGAGCGAGAUCGCCCACAGGUACAACGACCUGGGCGAGCAGCACUUCAAGGGCCUGGUGCUGAUCGCCUUCAGCCAGUAUCUCCAGAAGUGCAGCUACGACGAGCACGCCAAGCUGGUGCAGGAGGUGACCGACUUCGCCAAGACAUGUGUGGCCGACGAGAGCGCCGCCAACUGCGACAAGAGCCUGCACACCCUGUUCGGCGACAAGCUGUGCGCCAUCCCCAACCUGAGGGAGAACUACGGCGAGCUGGCCGACUGCUGCACCAAGCAGGAGCCCGAGAGGAACGAGUGCUUCCUGCAGCACAAGGACGACAACCCCAGCCUGCCCCCCUUCGAGAGGCCCGAGGCCGAGGCCAUGUGCACCAGCUUCAAGGAGAACCCCACCACCUUCAUGGGCCACUAUCUCCACGAGGUGGCCAGGAGGCACCCCUACUUCUACGCCCCCGAGCUGCUGUACUACGCCGAGCAGUACAACGAGAUCCUGACCCAGUGCUGCGCCGAGGCCGACAAGGAGAGCUGCCUGACCCCCAAGCUGGACGGCGUGAAGGAGAAGGCCCUGGUGAGCAGCGUGAGGCAGAGGAUGAAGUGCAGCAGCAUGCAGAAGUUCGGCGAGAGGGCCUUCAAGGCCUGGGCCGUGGCCAGGCUGAGCCAGACCUUCCCCAACGCCGACUUCGCCGAGAUCACCAAGCUGGCCACCGACCUGACCAAGGUGAACAAGGAGUGCUGCCACGGCGAUCUCCUGGAGUGCGCCGACGACAGGGCCGAGCUGGCCAAGUACAUGUGCGAGAACCAGGCCACCAUCAGCAGCAAGCUGCAGACAUGUUGCGACAAGCCCCUGCUGAAGAAGGCCCACUGCCUGAGCGAGGUGGAGCACGACACCAUGCCCGCCGAUCUCCCCGCCAUCGCCGCCGACUUCGUGGAGGACCAGGAGGUGUGCAAGAACUACGCCGAGGCCAAGGACGUGUUCCUGGGCACCUUCCUGUACGAGUACAGCAGGAGGCACCCCGACUACAGCGUGAGCCUGCUGCUGAGGCUGGCCAAGAAGUACGAGGCCACCCUGGAGAAGUGCUGCGCCGAGGCCAACCCCCCCGCCUGCUACGGCACCGUGCUGGCCGAGUUCCAGCCCCUGGUGGAGGAGCCCAAGAACCUGGUGAAGACCAACUGCGACCUGUACGAGAAGCUGGGCGAGUACGGCUUCCAGAACGCCAUCCUGGUGAGGUACACCCAGAAGGCCCCCCAGGUGAGCACCCCCACCCUGGUGGAGGCCGCCAGGAACCUGGGCAGGGUGGGCACCAAGUGCUGCACCCUGCCCGAGGACCAGAGGCUGCCCUGCGUGGAGGACUACCUGAGCGCCAUCCUGAACAGGGUGUGCCUGCUGCACGAGAAGACCCCCGUGAGCGAGCACGUGACCAAGUGCUGCAGCGGCAGCCUGGUGGAGAGGAGGCCCUGCUUCAGCGCCCUGACCGUGGACGAGACCUACGUGCCCAAGGAGUUCAAGGCCGAGACCUUCACCUUCCACAGCGACAUCUGCACCCUGCCCGAGAAGGAGAAGCAGAUCAAGAAGCAGACCGCCCUGGCCGAGCUGGUGAAGCACAAGCCCAAGGCCACCGCCGAGCAGCUGAAGACCGUGAUGGACGACUUCGCCCAGUUCCUGGACACAUGUUGCAAGGCCGCCGACAAGGACACAUGUUUCAGCACCGAGGGCCCCAACCUGGUGACCAGGUGCAAGGACGCCCUGGCCGGCGGCGGCGGCAGCGCCCCCACCAGCAGCAGCACCAGCAGCAGCACCGCCGAGGCCCAGCAGCAGCAGCAGCAGCAGCAGCAGCAGCAGCAGCACCUGGAGCAGCUGCUGAUGGAUCUCCAGGAGCUGCUGAGCAGGAUGGAGAACUACAGGAACCUGAAGCUGCCCAGGAUGCUGACCUUCAAGUUCUAUCUCCCCAAGCAGGCCACCGAGCUGAAGGAUCUCCAGUGCCUGGAGGACGAGCUGGGCCCCCUGAGGCACGUGCUGGACCUGACCCAGAGCAAGAGCUUCCAGCUGGAGGACGCCGAGAACUUCAUCAGCAGGAUCAGGGACACCGUGGUGAAGCUGAAGGGCAGCGACAACACCUUCGAGUGCCAGUUCGACGACGAGAGCGCCACCGUGGUGGACUUCCUGAGGAGGUGGAUCGCCUUCUGCCAGAGCAUCAUCAGCACCAGCCCCCAGUAAACUCGAGUGUUUUGGCUGGGUUUUUCCUUGUUCGCACCGGACACCUCCAGUGACCAGACGGCAAGGUUUUUAUCCCAGUGUAUAUUGUCGACAAAAAAAAAAAAAAAAAAAAAAAAAAAAAAAAAAAAAAAAAAAAAAAAAAAAAAAAAAAAAAAAAAAAAAAAAAAAAAAAAAAAAAAAAAAAAA |
| MOG27-63 | AGGCCGGCGGGUUUCUGACAUCCGGCGGGUUUCUGACAUCCGGCGGGUUUCUGACAUCCGGCGGGUUUCUGACAUCCGGCGGGUUUCUGACAUCCGGCGGGUUUCUGACAUCCGGCGGGUUUCUGACAUCCGGCGGGUUUCUGACAUCCGGCGGGUUUCUGACAUCCGGCGGGUUUCUGACAUUCACAACCAGGCCUCCACAACCAUGGCCGCCCCCGGCAGCGCCAGGAGGCCCCUGCUGCUGCUGCUGCUGCUGCUGCUGCUGGGCCUGAUGCACUGCGCCAGCGCCGCCAGCCCCGGCAAGAACGCCACCGGCAUGGAGGUGGGCUGGUACAGGAGCCCCUUCAGCAGGGUGGUGCACCUGUACAGGAACGGCAAGGACCAGGACGCCGAGCAGGCCCCCGGCCCCGGCCCCGGCAGCAGCGACUACACCAUCGUGCUGCCCGUGAUCGGCGCCAUCGUGGUGGGCCUGUGCCUGAUGGGCAUGGGCGUGUACAAGAUCAGGCUGAGGUGCCAGAGCAGCGGCUACCAGAGGAUCUAAACUCGAGUGUUUUGGCUGGGUUUUUCCUUGUUCGCACCGGACACCUCCAGUGACCAGACGGCAAGGUUUUUAUCCCAGUGUAUAUUGUCGACAAAAAAAAAAAAAAAAAAAAAAAAAAAAAAAAAAAAAAAAAAAAAAAAAAAAAAAAAAAAAAAAAAAAAAAAAAAAAAAAAAAAAAAAAA |
| p31 | AGGCCGGCGGGUUUCUGACAUCCGGCGGGUUUCUGACAUCCGGCGGGUUUCUGACAUCCGGCGGGUUUCUGACAUCCGGCGGGUUUCUGACAUCCGGCGGGUUUCUGACAUCCGGCGGGUUUCUGACAUCCGGCGGGUUUCUGACAUCCGGCGGGUUUCUGACAUCCGGCGGGUUUCUGACAUUCACAACCAGGCCUCCACAACCAUGGCCGCCCCCGGCAGCGCCAGGAGGCCCCUGCUGCUGCUGCUGCUGCUGCUGCUGCUGGGCCUGAUGCACUGCGCCAGCGCCUACGUGAGGCCCCUGUGGGUGAGGAUGGAGGGCCCCGGCCCCGGCAGCAGCGACUACACCAUCGUGCUGCCCGUGAUCGGCGCCAUCGUGGUGGGCCUGUGCCUGAUGGGCAUGGGCGUGUACAAGAUCAGGCUGAGGUGCCAGAGCAGCGGCUACCAGAGGAUCUAAACUCGAGUGUUUUGGCUGGGUUUUUCCUUGUUCGCACCGGACACCUCCAGUGACCAGACGGCAAGGUUUUUAUCCCAGUGUAUAUUGUCGACAAAAAAAAAAAAAAAAAAAAAAAAAAAAAAAAAAAAAAAAAAAAAAAAAAAAAAAAAAAAAAAAAAAAAAAAAAAAAAAAAAAAAAAAAAAAAA |

**Table S2. Range of physicochemical properties of used LNPs**

| **Ionizable lipid** | **Size [d.nm]** | **PDI** | **mRNA %eff** | **Zeta [mV]** |
| --- | --- | --- | --- | --- |
| ETG-23 | 71.4 – 86.57 | 0.040 – 0.122 | 93 – 100 | -0.41 – 7.68 |
| ETS-21 | 81.26 – 89.36 | 0.024 – 0.090 | 95 – 98 | -13.53 – -3.52 |
| MC-3 | 60.1–71.64 | 0.063 – 0.117 | 96 – 99 | -8.1 – 0.57 |
| SM-102 | 67.3 | 0.032 | 98 | 0.11 |

**Table S3. Antibodies used for cellular tropism**

| **Antibodies** | **Provider** | **Catalog Number** | **Mouse Panel** |  |
| --- | --- | --- | --- | --- |
| CD45 BUV496 | BD | 749889 | LIV1/LIV2/SPL |  |
| CD90.1 BV786 | BD | 740917 | LIV1 |  |
| CD80 BV421 | BD | 562611 | LIV1 |  |
| CD11b (M1/70) – BV605 | BD | 563015 | LIV1/LIV2/SPL |  |
| CD31 PE-Cy7 | Biolegend | 102418 | LIV1 |  |
| TIM4 PE | BD | 564147 | LIV1 |  |
| Ly6G (1A8) – PeCy5 | Biolegend | 127672 | LIV1 |  |
| Ly6C (HK1.4) – AF700 | Biolegend | 128024 | LIV1/SPL |  |
| PD-L1 (B7-H1) – APC | Biolegend | 124311 | LIV1 |  |
| CD3 PerCPeFluor710 | Invitrogen | 46-0032-82 | LIV2/SPL |  |
| CD4 (GK1.5) – BV421 | Biolegend | 100443 | LIV2 |  |
| CD19 (6D5) – BV785 | Biolegend | 115543 | LIV2/SPL |  |
| NK1.1 (NK1.1) – PE-eFluor610 | eBioscience | 17-0900-82 | LIV2/SPL |  |
| CD8a FITC | BD | 553031 | LIV2 |  |
| CD90.1 – APC | eBioscience | 17-0900-82 | LIV2/SPL |  |
| F4/80 (BM8) – eF450 | eBioscience | 48-4801-82 | SPL |  |
| CD11c BV650 | BD | 564079 | SPL |  |
| CD4 FITC | BD | 553729 | SPL |  |
| Ly-6G (GR1) – PE | eBioscience | 12-9668-82 | SPL |  |

**Table S4. Antibodies used for DC phenotyping**

| **Antibody** | **Supplier** | **Category number** |
| --- | --- | --- |
| Anti-CD3e (clone 145-2C11), BV605 conjugated | BioLegend | Cat# 100351 |
| Anti-CD19 (clone 6D5), BV605 conjugated | BioLegend | Cat#115539 |
| Anti-CD64 (clone ×54-5/7.1), BV711 conjugated | BioLegend | Cat#139311 |
| Anti-CD11c (clone N418), BUV496 conjugated | BD Biosciences | Cat#750450 |
| Anti-I-A/I-E (clone M5/114.15.2), BUV805 conjugated | BD Biosciences | Cat# 748844 |
| Anti-XCR1 (clone ZET), BV650 conjugated | BioLegend | Cat#148220 |
| Anti-CD172a (clone P84), PerCP-eFluor710 conjugated | Thermo Fisher Scientific | Cat#46-1721-82 |
| Anti-CCR7 (clone 4B12), PE-eFluor610 conjugated | Thermo Fisher Scientific | Cat# 61-1971-82 |
| Anti-CD11b (clone M1/70), AlexaFluor700 conjugated | BD Biosciences | Cat# 564985 |
| Anti-CD63 (clone NVG-2), PE-Cy7 conjugated | Thermo Fisher Scientific | Cat#25-0631-82 |
| Anti-F4/80 (clone BM8), BV785 conjugated | BioLegend | Cat#123141 |
| Anti-Ly-6C (clone HK1.1), BV570 conjugated | BioLegend | Cat#128029 |
| Anti-CD80 (clone 16-10A1), BUV737 conjugated | BD Biosciences | Cat#612773 |
| Anti-ICOSL (clone HK5.3), biotin conjugated | BioLegend | Cat# 107403 |
| Anti-CD86 (clone GL1), BV421 conjugated | BioLegend | Cat# 105032 |
| Anti-CD274 (clone 10F.9G2), AF488 conjugated | BD Biosciences | Cat# 568303 |
| Anti-CD103 (clone M290), BUV395 conjugated | BD Biosciences | Cat#568715 |
| streptavidin-PE-CF594 | BD Biosciences | Cat#562284 |
